# Context-based facilitation of semantic access follows both logarithmic and linear functions of stimulus probability

**DOI:** 10.1016/j.jml.2021.104311

**Published:** 2021-12-20

**Authors:** Jakub M. Szewczyk, Kara D. Federmeier

**Affiliations:** a Department of Psychology, University of Illinois at Urbana-Champaign, Champaign, IL, USA; b Donders Institute for Brain, Cognition and Behaviour, Radboud University, Nijmegen, the Netherlands; c Program in Neuroscience, University of Illinois at Urbana-champaign, Champaign, IL, USA; d Beckman Institute for Advanced Science and Technology, University of Illinois at Urbana–Champaign, Champaign, IL, USA

**Keywords:** Context-based facilitation, N400, Semantic access, GPT-2

## Abstract

Stimuli are easier to process when context makes them predictable, but does context-based facilitation arise from preactivation of a limited set of relatively probable upcoming stimuli (with facilitation then linearly related to probability) or, instead, because the system maintains and updates a probability distribution across all items (with facilitation logarithmically related to probability)? We measured the N400, an index of semantic access, to words of varying probability, including unpredictable words. Word predictability was measured using both cloze probabilities and a state-of-the-art machine learning language model (GPT-2). We reanalyzed five datasets (n = 138) to demonstrate and then replicate that context-based facilitation on the N400 is graded, even among unpredictable words. Furthermore, we established that the relationship between word predictability and context-based facilitation combines linear and logarithmic functions. We argue that this composite function reveals properties of the mapping between words and semantic features and how feature- and word-related information is activated on-line.

## Introduction

The 21st century marks a theoretical shift among brain researchers toward more fully appreciating how the processing of sensory information is shaped by internal models of the outside world. The study of human language processing provides a particularly fertile area for investigating how the brain builds and uses such internal models because language contexts offer rich opportunities to shape the manner and degree to which a given input word is predictable. There is a vast literature showing that comprehenders incrementally build mental representations of sentences and that these representations are exploited to facilitate the processing of upcoming words. The benefits stemming from contextual facilitation^1^ can be seen in both behavioral measures (e. g., reading times, eye movement behavior; Ehrlich & Rayner, 1981; McDonald & Shillcock, 2003; Traxler et al., 2000; Traxler & Foss, 2000) and brain responses (Baumgaertner et al., 2002; Dien et al., 2008; Hartwigsen et al., 2017; Lau et al., 2008), including facilitated event-related potential (ERPs) responses (Kutas & Hillyard, 1984; Van Berkum et al., 1999). In particular, the N400 component of the ERP, which has been linked to semantic access, shows a robust sensitivity to the fit between a word and its sentence context. Moreover, even though we focus here on words, the mechanisms indexed by the N400 extend to the match between an internal model and any meaningful stimulus, including pictures or scenes (reviewed in Federmeier, 2022; Kutas & Federmeier, 2011).

However, despite decades of work looking into how context information shapes processing, there are still critical gaps in our knowledge. One important question concerns the “scope” of context-based facilitation. On one view, which Brothers and Kuperberg (2021) refer to as the “proportional preactivation account,” words are preactivated in proportion to the statistics of the language environment. Contextual facilitation arises due to a match between the preactivated representations and the incoming stimulus, and the function relating word probability to facilitation should therefore be linear (for similar views, see also DeLong et al., 2005; Huettig & Mani, 2016; Kleinman et al., 2015; Reichle et al., 2003; Schwanenflugel & LaCount, 1988; Staub, 2015; Van Petten & Luka, 2012). Similarly, the computational model by Fitz and Chang (2019) assumes that one index of contextual facilitation, N400 amplitude, is directly linked to lexical representations in a linear way. The other view assumes that contextual facilitation effectively extends to all words in the lexicon and the amount of updating is equivalent to a word’s surprisal (the negative logarithm of probability), so it follows a logarithmic function (Aurnhammer & Frank, 2019; Kuperberg & Jaeger, 2016; Levy, 2008; Smith & Levy, 2013; see also Hale, 2001; Itti & Baldi, 2005 for precursors). Although various mechanisms have been proposed to underlie the logarithmic function, we will jointly refer to these proposals as the surprisal account.

The two types of views make different assumptions, and they differ along multiple dimensions (we will return to them in the General Discussion), but one clear, testable difference is in whether they posit a linear or a logarithmic relationship between word probability and contextual facilitation. The question of whether word probability has a linear or logarithmic relation to contextual facilitation will be the focus of the present study (with facilitation measured using the N400).

The most common way to assess a word’s probability in its context is simply to give people the context information (e.g., an incomplete sentence) and ask them to generate what they think will come next. The proportion of people who continue a particular context with a particular word is referred to as that word’s cloze probability. Cloze probability (CP) has proven to be a highly predictive metric both for behavioral responses (e.g., Duffy et al., 1989; Schuberth et al., 1981; Schwanenflugel & LaCount, 1988; Schwanenflugel & Shoben, 1985) and for N400 amplitudes (e.g., DeLong et al., 2005; Kutas & Hillyard, 1984). Studies that use CPs to measure contextual facilitation using the N400 have typically described the relationship as linear (DeLong et al., 2005; Kutas & Hillyard, 1984; Nieuwland et al., 2018), although few studies have formally assessed the shape of the function. Wlotko & Federmeier (2013) showed that, for words read in central vision, the addition of a quadradic term did not improve fit over the observed linear relationship between CP and N400 amplitudes^2^. Similar comparisons done in studies using reading times as an index of contextual facilitation have yielded mixed findings. Whereas Luke and Christianson (2016) showed that log-transformed CP provides a better fit to reading times, Brothers and Kuperberg (2021) reported that raw CPs are a better predictor. Also, as pointed out by Staub (2015), contrary to the idea that facilitation follows a logarithmic function, first-pass fixation times on truly anomalous words are not disproportionately longer than those to unexpected words that are not anomalous, as would be suggested by their (presumably) extremely low values on the log-probability scale.

The two accounts differ most in what they presume about how context affects the processing of words that are very unpredictable. For example, the surprisal-based account would predict a large effect when comparing two words with log-probability equal to ‒20 and ‒10, whereas, according to the linear account, the effect should be negligible, as the two words would differ only by 0.00005 on the linear scale. However, this is also where CP tests reach the limit of their utility because probability differences between unpredictable words are difficult or impossible to measure with production norms. If a context makes some word or small set of words predictable, people will always tend to produce those, even if other words are possible with varying levels of low predictability. In the case of very open-ended contexts, where no particular word(s) are likely to be brought to mind, production norms are limited by the fact that accurate measurement of the probability of all minimally predictable words would require extremely large samples of participants. For that reason, when using CP tests, one can effectively only compare the processing of moderately to highly probable words, i. e., those words that have a chance to occur in production norms.

As a way around these limitations, researchers have sometimes manipulated in a factorial way the degree to which improbable (or even incongruent) words fit their context. The level of fit was most often estimated using plausibility, for example, based on ratings from a normative group of participants. Sometimes the degree of fit was also manipulated by the presence or absence of a contextually supported semantic feature (e.g., animacy) or by the words’ general overlap with the event or schema elicited by the context^3^. These studies have yielded mixed results. For example, some studies have found that N400 amplitudes are sensitive to these manipulations (Brothers et al., 2020, experiment 2b; DeLong et al., 2014; DeLong & Kutas, 2020; Kuperberg et al., 2020; Lau, Namyst, et al., 2016; Quante et al., 2018, contrast between unexpected plausible and anomalous; Stites et al., 2017; Vega-Mendoza et al., 2021) but others have not (Brothers et al., 2020, experiment 2a; Hagoort et al., 2004; Holcomb et al., 1999; Kuperberg et al., 2010; Quante et al., 2018, contrast between anomalous possible and impossible; Shetreet et al., 2019; Szewczyk & Schriefers, 2011; van de Meerendonk et al., 2010; Zhang et al., 2012). Because plausibility is generally estimated based on subjective, off-line assessments, which can thus be affected by decision thresholds and other processes that may occur subsequent to the effects of contextual facilitation, these approaches may be less able to reveal the degree of facilitation a word is likely to receive during online processing (Kutas & Federmeier, 2011).

All in all, to understand the nature of the relationship between word probability and context-based facilitation—a core piece of information for delineating the mechanisms at work—we need to find some way to reliably assess the probability of words that will not appear in cloze production norms and, ideally, to do so in a way that does not depend upon subjective ratings. A potential solution is to assess word predictability by probing machine learning algorithms instead of using cloze tests conducted with human subjects. Language models allow us to, for any position in the sentence, estimate the probability distribution across all words in a lexicon, not only those that are likely for humans to produce. Moreover, in contrast to human subjects, these algorithms by design can differentiate extremely low probabilities. With the advent of attention-based algorithms, and the Transformer architecture in particular (Vaswani et al., 2017), models have started to excel on a variety of language tasks, such as coreference resolution, summarization or question answering (in fact, they currently dominate all top benchmarks in the field of natural language processing; Rajpurkar et al., 2018; A. Wang et al., 2019). Some of these models have also demonstrated the ability to produce coherent paragraphs of text that often, at least superficially, are not distinguishable from text generated by humans (Radford et al., 2019)—an important, albeit very informal, way of determining that the models are good at estimating the predictability of words.

Prior work using language models to predict the N400 and other indices of context-based facilitation usually assumed a logarithmic relationship, and thus the majority of work used surprisal as the predictor (e.g., Boston et al., 2008; Brennan et al., 2016; Demberg & Keller, 2008; Frank et al., 2015; Frank & Bod, 2011; Heilbron et al., 2021; Hu et al., 2020; Merkx & Frank, 2020; Monsalve et al., 2012; Schmitt et al., 2020; Shain et al., 2020; van Schijndel & Linzen, 2018; Wilmot & Keller, 2020). Moreover, some of these studies either presented data that were in line with the assumption of logarithmic relationship (Goodkind & Bicknell, 2018; Wilcox et al., 2020) or explicitly compared the fit of logarithmic and linear models to show the superiority of the former (with probabilities estimated using a 3-gram model: Smith & Levy, 2013; or a 5-gram model: Yan & Jaeger, 2020). However, that work can be also criticized on the grounds that it relies on the assumption that cloze probability and probability estimated by language models is measured on the same scale. Thus, it is crucial first to establish if probabilities for the same set of words estimated by CP tests and by language models produce comparable shapes of relationship with brain and behavioral data. An additional limitation of the studies that directly compared the linear and logarithmic functions is that they are based on ngram models, which are currently outdated and are known to generate inferior estimates of word probability (see below). This may be a source of both bias and of noisier estimation which becomes critically important for distinguishing two quite similar functions (see Brothers & Kuperberg, 2021 who show that underpowered studies may easily lead to the wrong selection between linear and logarithmic functions).

Finally, most of the studies looking at the relationship between predictability and context-based facilitation have measured facilitation behaviorally. However, this may be not ideal because language comprehension has no necessary behavioral correlate, so studies using response times need to introduce a secondary task. Response time measures thus reflect not only context-based facilitation but also other processes related to the task itself, and therefore may be biased. Eye movement measures have the advantage of being a natural correlate of normal reading, yet, as end state measures, they also summate across multiple aspects of processing. In contrast, ERPs provide a means of tracking comprehension online, without requiring additional tasks, and the N400 has been closely linked with semantic access processes in particular (Federmeier, 2022; Kutas & Federmeier, 2011). Thus, N400 measures afford a focused assessment of the impact of context-based probability on the target of theoretical interest – the online semantic processing of an incoming word.

## Current study

In this study we focus on estimating the shape of the function that relates word probability to context-based facilitation—and, by extension, to probe whether contextual facilitation is or is not limited in scope. We will measure the shape of the function, as in some previous work, but do it in a way that goes beyond human CPs, using language models to get better estimates of the probabilities of words that have measured CPs at or near 0—which make up almost half of our data sample. We focus in particular on that part of the range because it is the part we know the least about and that carries the most weight for determining the scope of facilitation. If the N400 to unexpected words shows substantial variance as a function of log-probability, then it should be concluded that the view assuming unbounded scope of context-based facilitation is correct. If, conversely, indices of word probability explain no or little variability in N400 amplitude to unexpected words, this will support the limited-facilitation views.

To this aim, we will obtain probability estimates not only for expected words, for which we also have CPs from humans, but also for unexpected words (which, as discussed, are difficult or impossible to get from CP measures) by using a Transformer language model trained to predict the next word (GPT-2, Radford et al., 2019). Transformer is a type of natural language model architecture. It is now widely used in real-life applications. Crucially for us, the next-word probability estimates produced by these models was shown to be an impressive predictor of a host of indices of behavioral performance and brain activation, surpassing preceding generations of neural network models (reading times: Merkx & Frank, 2020; Wilcox et al., 2020; fMRI data: Schrimpf et al., 2020; MEG data: Caucheteux & King, 2020; N400 amplitudes: Merkx & Frank, 2020; Heilbron et al., 2021; ECOG activity: Goldstein et al., 2020; Schrimpf et al., 2020). Importantly, the improvement in linguistic accuracy (i.e., how good a model is at predicting the next word) converges with the model’s “psychological” accuracy, i.e., how well it predicts context-based facilitations as measured in reading behavior and in the brain (Caucheteux & King, 2020; Goldstein et al., 2020; Schrimpf et al., 2020). Moreover, it is particularly the model’s accuracy in the next-word prediction task (and not any other index of the model’s performance) that explains its success in modeling brain activity (Schrimpf et al., 2020), which may indicate that—at least at the computational level—the brain pursues the same objective as the models trained in the next-word prediction task.

As a means of validating the measure, we will for the first time compare the model’s ability to predict N400 amplitudes against predictions based on human-generated CPs—which we know are a good predictor of N400s, for the part of the range of probabilities where CPs are above zero (i.e., expected words). This will enable us to test whether the model’s predictions are a good proxy of human production norms. Assuming they are, then, we will use the language model to explore the part of variance in contextual fit that has been heretofore intractable and see whether N400 amplitude to unexpected words—which all had CPs near or at 0—shows substantial variance as a function of log-probability. This will help us explicate the mechanisms at play and, in particular, whether the brain’s sensitivity to context affects the processing of words beyond the set that are likely to be explicitly produced. To conduct these analyses, we used data from previously published experiments in which native speakers of English read simple sentences while their EEG was recorded. The sentences ended with more or less expected words. The analysis was done in two steps. We first focused on one dataset, and then confirmed and replicated the findings on four other datasets.

## Method

In this first part of the study, we reanalyze data which were previously reported in Federmeier et al. (2007).

### Participants

Thirty-two right-handed native English speakers (sixteen female) at the University of California, San Diego participated in the study in exchange for course credit or cash (mean age 20, range 18–28). All participants were right-handed by self-report and as assessed by the Edinburgh inventory (Oldfield, 1971). Seven participants reported having left-handed or ambidextrous family members. All participants reported normal vision and none had a history of neurological or psychiatric disorders.

### Materials

The experimental stimuli included 282 sentences. Half of the sentence endings were best completions (the highest CP word for that context) whereas the other half were alternate completions that were of very low CP. These sentence endings served as the critical words. Several experimenters (from the original study by Federmeier et al., 2007) judged the unexpected endings to be plausible in their sentence frame and to come from a different semantic category from (and thus share relatively little feature overlap with) the corresponding expected ending. Mean association strength (as assessed by the Edinburgh Associative Thesaurus; approximately 90% of the experimental stimuli were in the database) between the expected and unexpected endings for each sentence frame was less than .005. Moreover, the endings were not associated with any word in the corresponding sentence frame (<2% of items had at least one moderate to strong associate, i.e. .2 or greater).

Sentences were divided into two lists, such that each participant saw each sentence frame only once; within each list, half of the frames for each constraint level were completed by the expected ending and half were completed by the unexpected ending (yoked so that the matched unexpected endings did not appear in the same list). The order of sentence frames was randomized once for each list and then presented in the same order to each participant.

#### Cloze test norming

CP of the endings was determined in a norming procedure with native English speakers at the University of California, San Diego (none of whom participated in the main ERP experiment). Each sentence frame was presented to at least 18 participants who were asked to read each sentence frame and to write down the word they “would generally expect to find completing the sentence fragment.” In an extension of the standard procedure, the instructions directed participants to give two additional plausible completions (however, CP calculation was based on the first completions in this study). Based on the norming, we defined expected endings as those with CP > = .05 (mean CP = .54, SD 0.32, range .05–1.00, 301 endings) and the rest as unexpected endings (mean CP = .005, SD 0.01, range 0–.05, 263 endings).

#### Estimates of Endings’ predictability based on a neural network

To estimate the predictability of sentence endings we used GPT-2 architecture, a decoder-only variant of the Transformer deep neural network architecture (Radford et al., 2019). This model has been trained at the next-word prediction task. It can provide the probability of any word conditional on a preceding context. We used the GPT2-xl variant (48 layers, 25 self-attention heads each, embedding size 1600, 1558 M parameters overall), and with weights pre-trained by OpenAI using the WebText corpus. The model generates predictions at a sub-word level, using the byte-pair encoding (BPE). The probability of words consisting of more than one chunk was computed using the chain rule. We added a period to each tested word to cue the model that it is the last word of the sentence, mimicking the experience gained by participants of the main experiment (and cloze test norming) after a few items. On an independent dataset (Szewczyk et al., 2021) we confirmed that doing so increases the psychometric predictive power of obtained probability estimates for words that occur as sentence endings. To obtain the context-based words’ predictabilities we modified the original Tensor-Flow code (https://github.com/openai/gpt-2) to generate chunk probabilities. We also altered the GPT2-based probabilities to eliminate artifacts introduced when BPE chunks are used to estimate word predictability. Such situations occur when a word consists of two or more chunks and the first one (or more) are also words (e.g., “ crowbar” consists of two BPE chunks: “ crow”+”bar”), which leads to artificial inflation of the probability of the constituent chunks (e.g. “ crow”).

#### Plausibility ratings

Each sentence and each ending was rated for expectancy and plausibility by 23–36 (mean: 31) participants recruited through Amazon Mechanical Turk. The sentences were intermingled with stimuli from other experiments (reported later) and appeared in several different lists. Each list contained a mix of less and more plausible items in order to encourage a full use of the rating scale from completely expected and plausible to entirely unexpected and implausible. Participants were asked to rate, on a scale of 1–7, how expected each sentence ending was and, in a separate judgment, how plausible is the ending of the sentence. A rating of 7 indicated very expected or completely plausible, and a rating of 1 indicated totally unexpected or completely nonsensical. Examples were given to attempt to draw participants’ attention to the difference between expectancy and plausibility. Split-half reliability of plausibility estimation was satisfactory (median r = .91 across 100 random splits of the data). In this analysis, we only used plausibility but not expectancy ratings.

### Procedure

Participants were seated 100 cm in front of a 21^′′^ CRT monitor. Each trial began with a warning sign (several pluses on the screen) presented for 500 ms; the blank screen between the warning sign and the first word of the sentence varied randomly from 500 to 1200 ms (to prevent the consistent buildup of anticipatory slow-wave activity). Sentences were then presented word by word in the center of the screen. Words were presented at the rate 2 per second (200 ms on, 300 ms blank screen). A 3-s pause separated each sentence.

Participants were asked to minimize blinks, eye movements, and muscle movement while reading. They were instructed to read for comprehension and told that they would be asked questions about what they had read at the conclusion of the recording session. The recording session began with a short set of practice sentences to acclimate the participants to the task situation. The main experimental session was divided into four blocks of sentences, with participants taking a short rest between each block; recording time was approximately 1 h.

After the recording session ended, participants completed a recognition test. A list of 240 words was selected such that, for each participant, 80 of the words were never seen as sentence-final words during the experiment, and, of the remaining 160 words, 40 sentence-final words came from each experimental condition. Participants were asked to circle all the words that they remembered seeing as a final word of one of the sentences in the experiment.

### EEG recording and preprocessing

EEG was recorded from 26 electrodes arranged in an equidistant montage (see Appendix A for electrode layout). Recordings were referenced to the mean of the left and the right mastoids. Additional electrodes were placed on the outer canthus of each eye to monitor horizontal eye movements, and over the left infraorbital ridge to monitor for vertical eye movements and blinks. Electrode impedances were kept below 5kΩ. Off-line, the continuous recordings were filtered with a high-pass zero-phase FIR filter at 0.1 Hz, transition band 0.1–2 Hz.

We analyzed epochs from the continuous EEG in the interval between ‒100 and 800 ms with respect to the onset of the target word (sentence ending). Systematic artifacts resulting from eye movements, blinks, and artifacts resulting from poor electrode contact were filtered out using AMICA (Palmer et al., 2011) run on 1-Hz-filtered data restricted to periods when the sentences were displayed. We first removed all trials containing horizontal eye-movements detected using an individualized threshold on a step function convoluted with the ICA channels corresponding to horizontal eye-movements. Segments and electrodes that had remaining artifacts (skin potentials, occasional poor electrode contact, etc.) were rejected using a logistic regression-based algorithm trained on manually marked artifacts (the algorithm and weights were the same as used in Szewczyk & Wodniecka, 2020). On average, 12% of segments were rejected. Instead of doing classical subtraction-based baseline correction, we regressed out the mean 100 ms pre-stimulus baseline amplitude in the statistical analyses (Alday, 2019). All preprocessing (except ICA) was coded in R using the eeguana package (version 0.1.5.9; Nicenboim, 2018).

### Statistical analyses

In all analyses, we were interested in fitting the amplitude of the N400 component, defined as the mean amplitude at four centro-parietal electrodes (MiCe, MiPa, LMCe, RMCe) in the 300–500 ms time-window. The analysis was performed stepwise. First, we focused on expected items, on which we validated predictability estimates made by the GPT2 model against CPs. The main analyses consisted in comparing three mixed-effects linear regression models. Each of the models predicted N400 amplitude to expected sentence endings, based on CP, GPT2, or both estimates of predictability considered together. The models also included covariates intended to statistically adjust for lexical properties of the words and their position in the sentence: lexical (log)frequency (Brysbaert & New, 2009), lexical neighborhood (OLD20; Yarkoni et al., 2008), concreteness (Brysbaert et al., 2014), and position relative to sentence onset. As indicated earlier, each model also contained the pre-stimulus baseline as a predictor. All predictors were centered and covariates were also normalized. Each model had an identical random-effects structure including by-subject and by-item intercepts, by-subject random slopes for the baseline, the two indices of predictability, and all covariates, and a by-item random slope for the baseline. In this and all further analyses we use a Gaussian likelihood, as the N400 effects are typically normally distributed. The model did not include correlations between random effects. We compared the two simpler models against the model including both indices of predictability using likelihood ratio tests. We also wanted to test whether linear or logarithmic predictability better fits the N400 data. To this end, we compared a linear, logarithmic, and a linear + logarithmic model for predictability measured using CPs and GPT2. Also here, the more complex model was compared against the two simpler models. To visually explore potential nonlinearities in the association between N400 amplitude and the linear and logarithmic predictability, we fitted Generalized Additive Models (GAMs). The GAMs had the same structure as the linear and logarithmic mixed-effects models, but with added cubic regression spline terms for the baseline and either the linear or logarithmic predictability.

Next, we focused on unexpected words. In this analysis, we only used logarithmic predictability estimated using GPT2. To test whether unexpected sentence endings’ predictability explains N400 variance, we fitted a linear mixed-effects model with the pre-stimulus baseline, predictability, and all covariates as predictors (and random effects structure analogous to that used in the analyses on expected words). We also explored this model visually using GAMs (adding cubic spline regression terms for all predictors).

Then, we tested to what extent unexpected sentence endings’ plausibility ratings can serve as a replacement for GPT2-estimated predictability in estimating the N400. We compared three linear mixed-effects regression models, including just plausibility, just predictability, or both predictors. Each model also included the pre-stimulus baseline, all covariates, and random effects structure analogous to the previous models. We compared the more complex model with the two simpler models using the likelihood ratio tests. The analyses described above were followed up with an additional exploratory set of analyses, which are described in the Results.

### Data and code availability

We report all data exclusions, all manipulations, and all materials in the study. Analysis code and research materials are available at https://osf.io/urvax. This repository also contains N400 averaged data necessary to reproduce all analyses (except point-by-point analyses) and figures reported in this paper. Preprocessed segmented EEG data (necessary to reproduce the point-by-point analyses) are available via Harvard Dataverse repository at https://doi.org/10.7910/D VN/ICLMHD, contingent on signing a ‘Terms of use’ agreement. The analyses reported here were not preregistered. In this paper, we used the following R packages: data.table (Dowle & Srinivasan, 2021), eeguana (Nicenboim, 2018), eegUtils (Craddock, 2019); stringr (Wickham, 2019), dplyr (Wickham et al., 2021), lme4 (Bates et al., 2015), brms (Bürkner, 2017), loo (Vehtari et al., 2020), mgcv (Wood, 2017), itsadug (van Rij et al., 2020), ggplot2 (Wickham, 2016), RColorBrewer (Neuwirth, 2014), patchwork (Pedersen, 2020), ggridges (Wilke, 2021), scales (Wickham & Seidel, 2020), doParallel (Microsoft Corporation & Weston, 2020), car (Fox & Weisberg, 2019). See Appendix E for versions of the used software.

## Results

### Correlations between estimates of word probability from cloze tests and from the GPT2 model

**Fig. 1.**
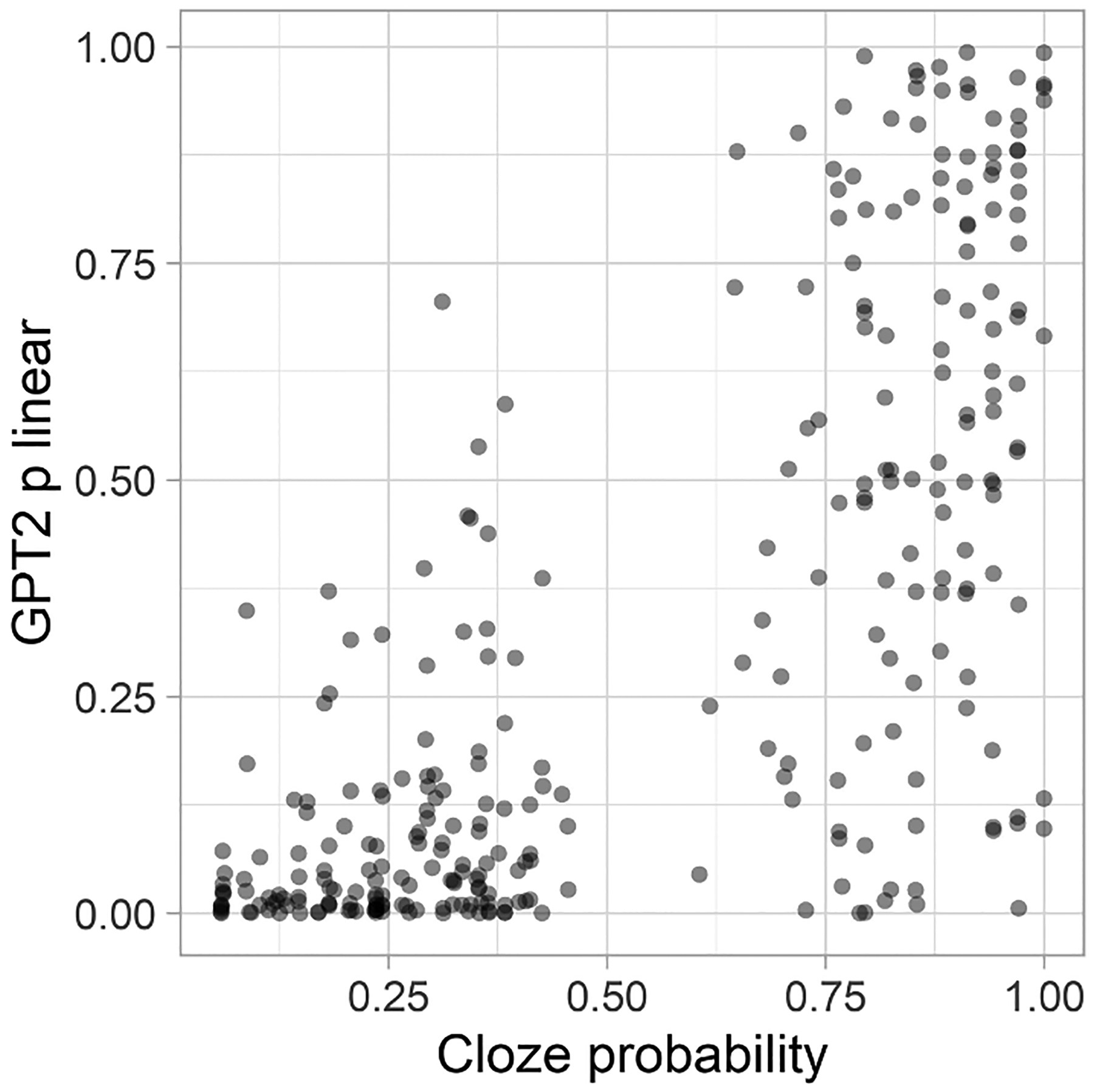
Scatterplots showing the relationship between two indices of word probability given context: cloze probability and (linear) GPT2 probability.

We began by examining the extent to which probabilities (on the linear scale) derived from GPT2 are comparable to human CP measures. We focused on the expected endings because in the case of unexpected words CPs have almost no variance. Pearson correlation coefficients between CPs and GPT-derived indices of predictability showed that these two predictors correlated significantly (r = .72; CI = .67–.77; p < 10^−16^; see Fig. 1). This correlation coefficient closely replicates an estimate from Goldstein et al. (2020)—r = .79—which was based on words embedded in short stories. The scatterplot suggests that the largest discrepancies between GPT2 and cloze norms occur for high-CP words: Not all words with high CP were considered to be highly probable by the language model. However, words that were infrequently produced as sentence continuations in the CP tests were also considered less probable by the GPT2 model.

### Predicting N400 amplitudes to expected words

In this set of analyses, we wanted to test what shape better explains the relationship between word predictability and the N400 to expected words: linear or logarithmic. We also wanted to test if CP tests and the GPT2 language model estimate word predictability on the same scale (for example, if the relationship between CP and N400 is linear, will the relation of GPT2 and N400 also be linear?). Finally, we tested if CP or GPT2 is a better predictor of N400 amplitudes to expected words.

**Fig. 2.**
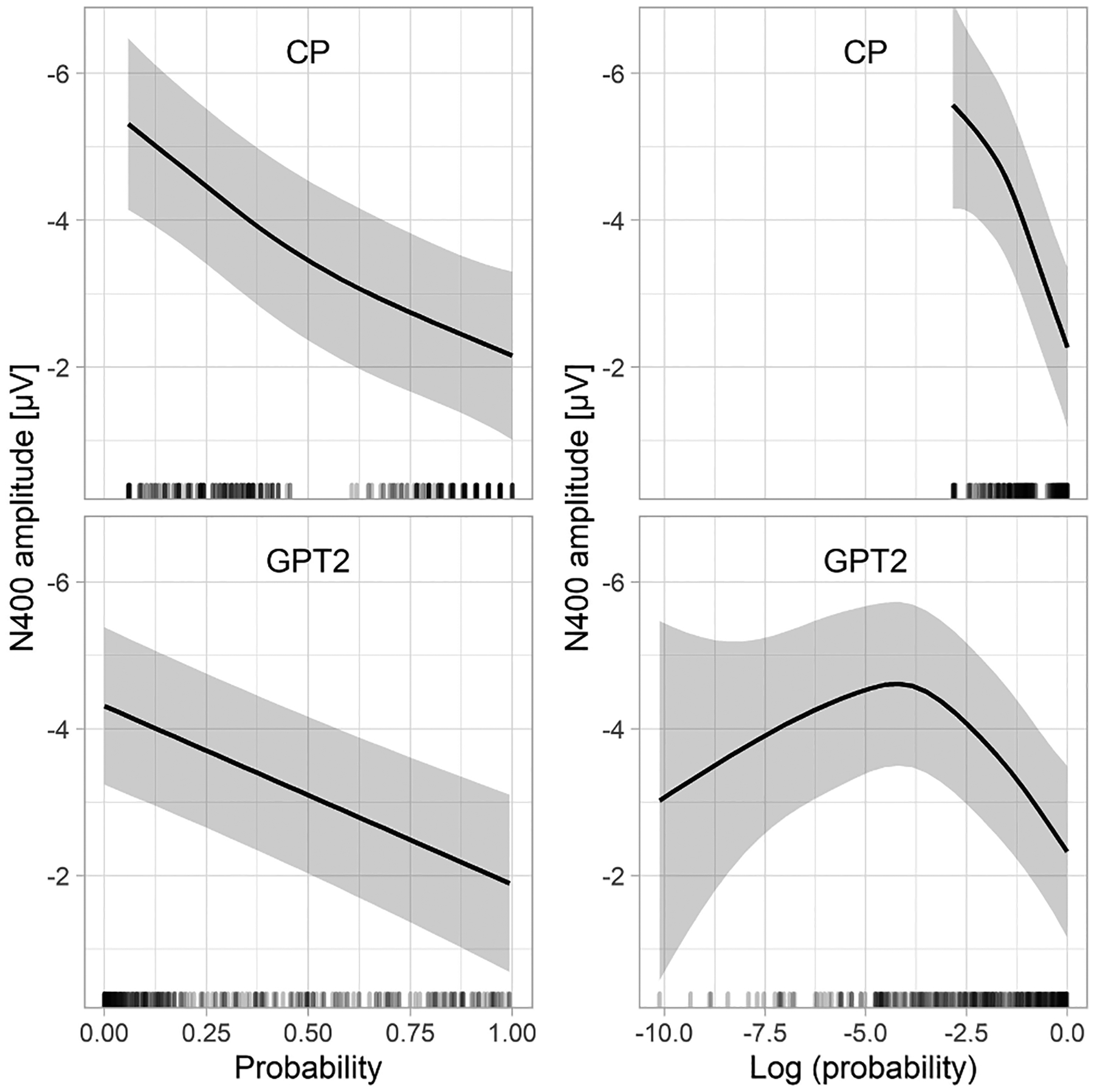
The Relationship Between Different Estimates of Word Probability and N400 amplitude to expected words, as estimated using Generalized Additive Models. Note. Top panels: Cloze Probability (CP); bottom panels: GPT2 probability; left panels: linear scale; right panels: logarithmic scale (surprisal). The shaded areas indicate 95% confidence intervals. The lines at the bottom of each panel show probabilities of individual items.

To this aim, we made three groups of model comparisons. The first one compared the fits of models based on CP on the linear and logarithmic scale (Table 1, top 2 rows). These comparisons show that neither linear nor logarithmic CP explains N400 variance over and above the other predictor, even though, numerically, linear CP gives a better fit to the data than logarithmic CP. The results are more clear-cut for the GPT2 model (Table 1, middle 2 rows). It shows a clear advantage of the linear version of the predictor over the logarithmic version. This can be also appreciated in Fig. 2 showing the relationship between CP/GPT2 probability and the N400 estimated using non-linear regression (GAM). When linear predictors are used, they lead to a linear relationship with N400 amplitude. Conversely, when logarithmic predictors are used, they lead to a nonlinear relationship with the N400, suggesting that the linear version provides a better fit to the data.

Next, we compared whether linear CPs or linear GPT2-estimated probabilities better predict N400 amplitudes to expected words. The model comparisons showed that CP explained additional variance on top of GPT2 linear probability, but not vice versa (see the bottom section of Table 1). According to the model using CPs, N400 amplitude to expected words as a function of word probability spans a range of about 2.5 μV (see also Fig. 4, left panel).

In summary, both CPs and probabilities estimated from the GPT2 language model seem to be linearly related with the N400 amplitude to expected words, across the full range of constraint (i.e., where CP of the items ranges from 0.05 to 1.00). Both CP and GPT2-derived probability provide a good estimate of the N400, but probabilities derived from human production norms outperform those estimated by the language model. Importantly, both indices seem to measure probability on the same scale, which warrants their interchangeability in future studies.

### Predicting N400 amplitudes to unexpected words

Next, we turned to the critical question: Is there variance in N400 amplitudes to unexpected words that can be accounted for by graded word probability? In these analyses we entirely relied on log-probabilities estimated using the GPT2 model because CPs for almost all unexpected words was equal to 0 and thus did not vary. It would also make no sense to use GPT2-derived probabilities on the linear scale, as for unexpected words such a predictor would have almost no variance.

**Fig. 3.**
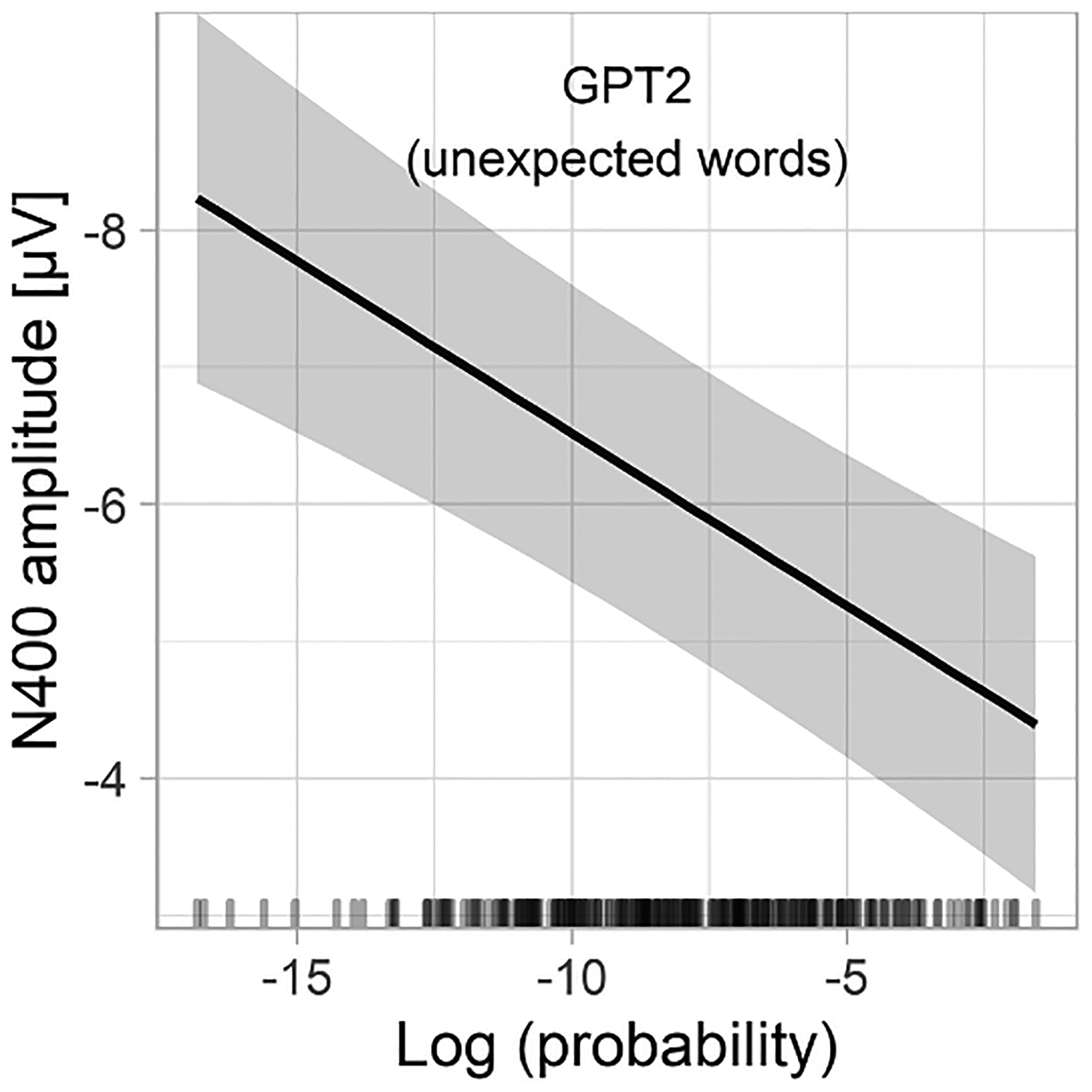
Output of a GAM model testing the relationship between log GPT2 word probability and the N400 to unexpected sentence endings.

**Fig. 4.**
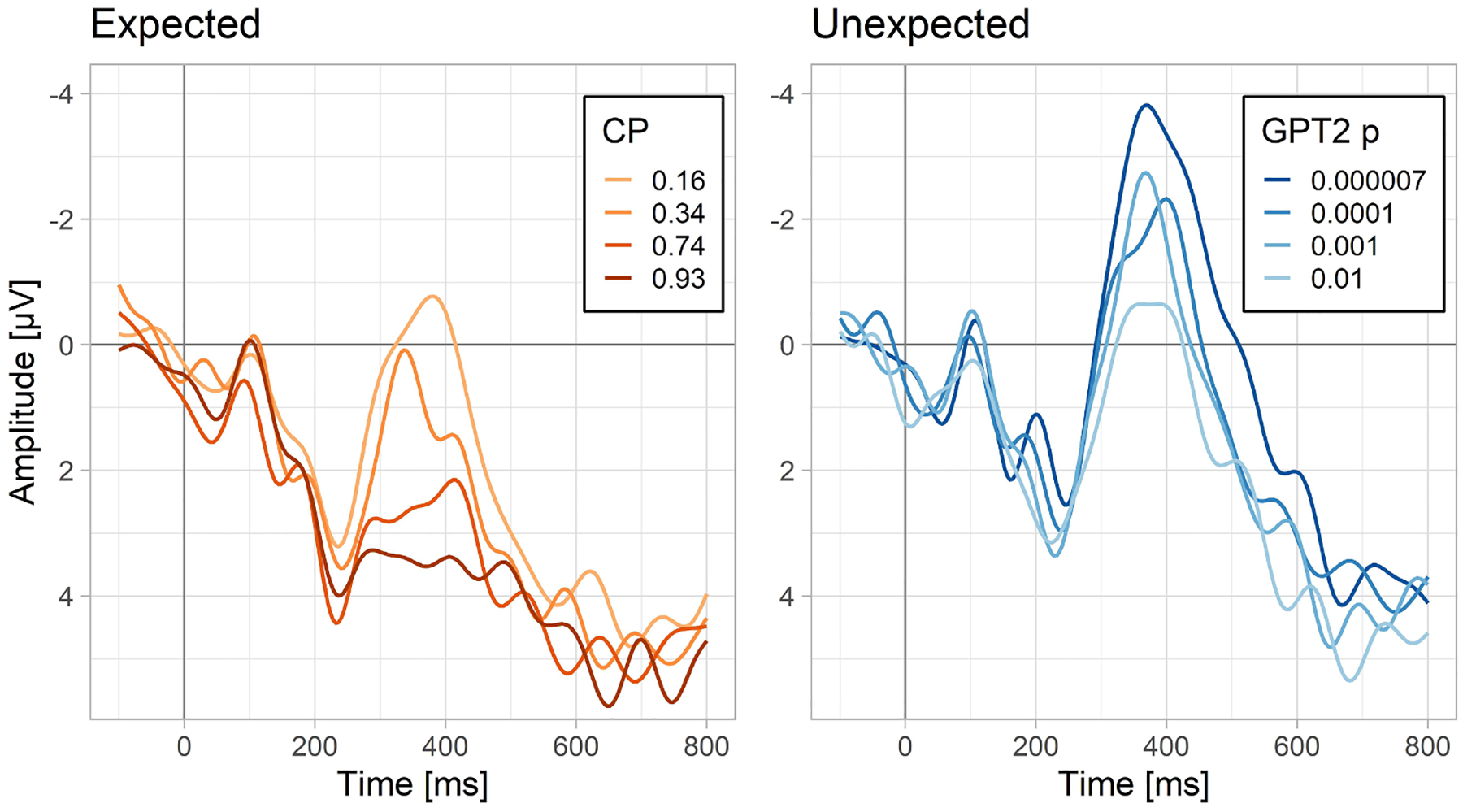
ERPs to Expected and Unexpected Sentence Endings. Note. ERPs were recorded at a midline parietal electrode (MiPa). Left panel: expected endings; right panel: unexpected endings. The ERPs are broken down by target word probability grouped into four bins with an equal number of elements, estimated using CP tests (left panel) and GPT2 log-probability (right panel; values in the legend were exponentiated to linear probability). For display purposes, the ERPs were low-pass filtered at 12 Hz.

To address this question, we fitted a linear mixed-effects model including GPT2 log probability as a predictor and the N400 amplitude to unexpected words as the dependent variable. The analysis showed a robust effect of word predictability (t = 5.1; see Appendix B for model details). Fig. 3 shows the predictions of a non-linear version of the same model (which for illustrative purposes was fitted with a statistical model allowing for non-linearities, i.e., Generalized Additive Model). The relationship between the GPT2 log probability (surprisal) and N400 to unexpected words shows no signs of a non-linearity, which suggests that the relationship is strictly linear (or, in other words, that the relationship between probability and N400 to unexpected words is strictly logarithmic). This model predicts that for the data at hand, the N400 amplitude to unexpected words spans a surprisingly wide range (>4 μV) as a function of GPT2 log probability. This can be also appreciated in Fig. 4 which shows a direct comparison between the ERPs evoked by expected and unexpected words varying in their probability on the linear and logarithmic scale, respectively. This figure also confirms that there is a continuity in the N400 amplitudes between expected and unexpected words (i.e., the N400 amplitude to the least probable expected word is similar to the N400 amplitude to the most probable unexpected word).

### Using plausibility ratings to predict the N400 amplitude to unexpected endings

As overviewed in the introduction, some research has probed for variance in behavioral or neural indices of contextual facilitation to unexpected words using plausibility ratings. Our analyses using model-estimated probabilities demonstrated that there is a lot of explainable variance in N400 amplitude to unexpected items. Here we examine whether the same variance can be explained using human plausibility ratings. Note that the two predictors—plausibility and GPT2-based log-probability—were correlated to some degree (for unexpected words, r = .39, see appendix G for scatterplots and an analogue of Fig. 4 with plausibility instead of log-probability), so it is likely that plausibility can explain some variance in N400 amplitudes even if just through the variance shared with word log-probability.

First, we ran a GAM mixed-effects model to explore whether the relationship between rated plausibility and N400 amplitude to unexpected items is linear. The model (presented in Appendix C) reveals that there is no support for a non-linear relationship (smoothing term p = .99). To compare the explanatory power of plausibility and GPT2 log-probability, we compared a full LMER model including both predictors and two nested models with either of the two predictors removed (and, as previously, baseline terms as well as covariates). The model comparison showed that GPT2 log probability is a better predictor of N400 amplitude to unexpected words (ΔlogLik = 7.6; p < .0001), whereas plausibility does not reliably explain additional variance above GPT2 log-probability (ΔlogLik = 1.9; p = .054).

### Characterizing N400s to expected and unexpected words using one function

In the analyses above we made a split between unexpected and expected words at an arbitrary point in the word probability continuum. This split seemed to differentiate the shape of the relationship between word probability and N400 into a linear and a logarithmic part, implying that the language comprehension system may be differently sensitive to probability at different regions of the probability scale. Here we followed up on this finding and asked if it is possible to construct a function that accurately expresses this sensitivity across the entire range of word probability. The simplest way to do this was to combine the logarithmic and linear functions. Such a function can have a predominantly linear shape in the highest probability region where probability on the linear scale is markedly away from zero, while maintaining the logarithmic shape when probability on the linear scale is very close to zero. This led to an alternative regression formula that could be used to fit data including both expected and unexpected words: N400 = *β*_1_*log(p) + *β*_2_*p + *ε*. Coefficients β_1_ and β_2_ control the steepness of the logarithmic and linear parts, whereas their proportion determines the breaking point between the logarithmic and linear part.

**Fig. 5.**
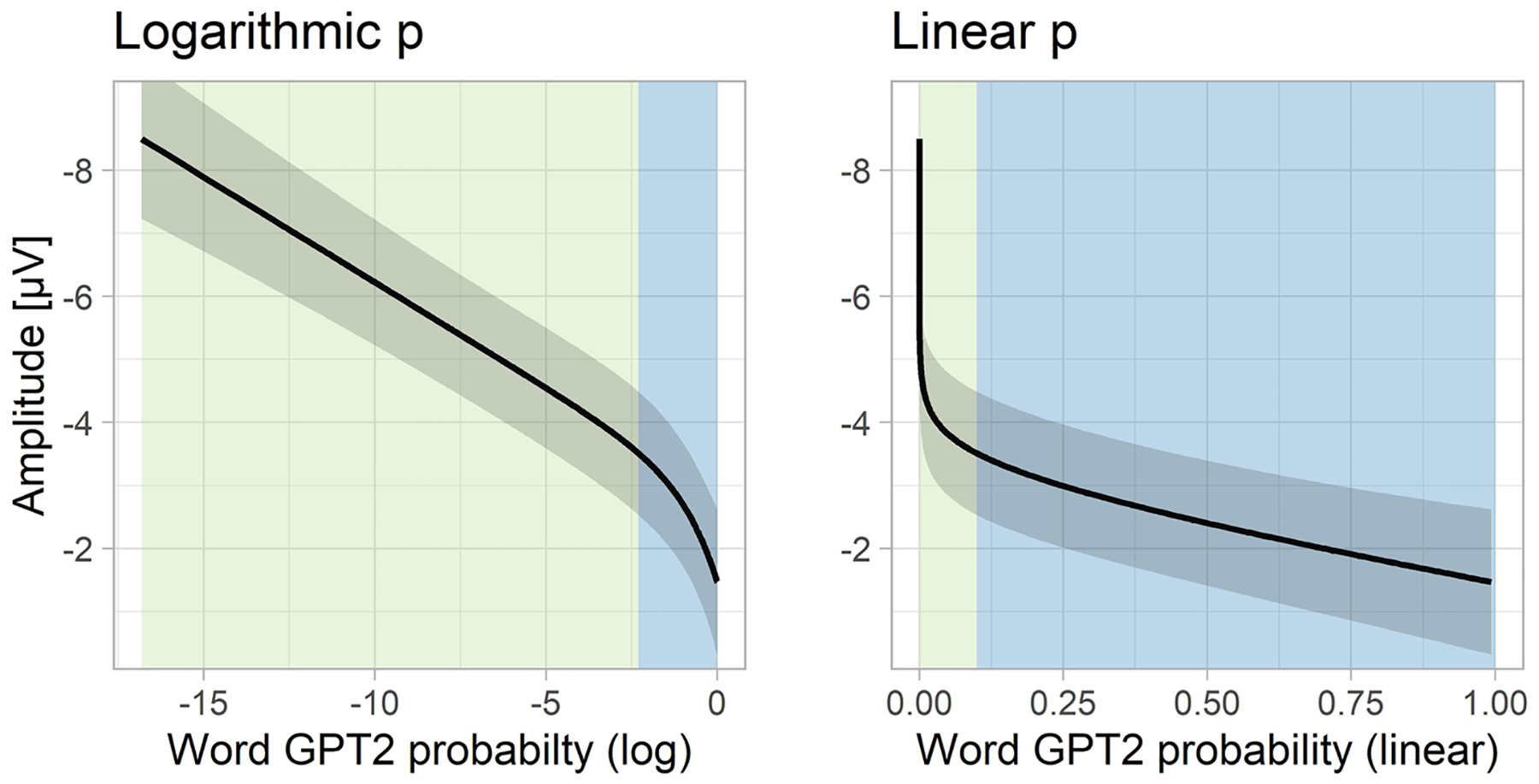
Predictions of the best-fitting model of N400 amplitude. Note. The model includes predictors of word probability on both the linear and logarithmic scale. The grey shaded area corresponds to a 95% confidence interval. Left panel: probability on the logarithmic scale; right panel: probability on the linear scale. The two background colors correspond to a threshold (arbitrarily set at p = .1) delineating regions where the relationship between word probability and N400 is more linear and more logarithmic.

To verify if the composite function is a better descriptor of N400 amplitudes than either a strictly linear or strictly logarithmic function for the dataset consisting of both expected and unexpected words, we compared a maximal model, containing both probability-related predictors (with all random slopes but no correlation between them) with two simpler models only consisting of the fixed effect of word probability on the linear scale or on the logarithmic scale. In all three models, the estimate of probability was based on the output of the GPT2 language model (so that we could maintain a consistent measure across the full range of word likelihood). Model comparison showed that both the linear (ΔlogLik = 3.9, p < .01) and the logarithmic (ΔlogLik = 23.2; p < .000001) components significantly contributed to the model’s fit to the data on top of the other predictor. Fig. 5 shows the empirically-determined probability function that incorporates both the linear and the logarithmic components, and Table 2 shows the summary of the model producing these values.

### Time-course and spatial distribution of the linear and the logarithmic subcomponents of the N400 effect

**Fig. 6.**
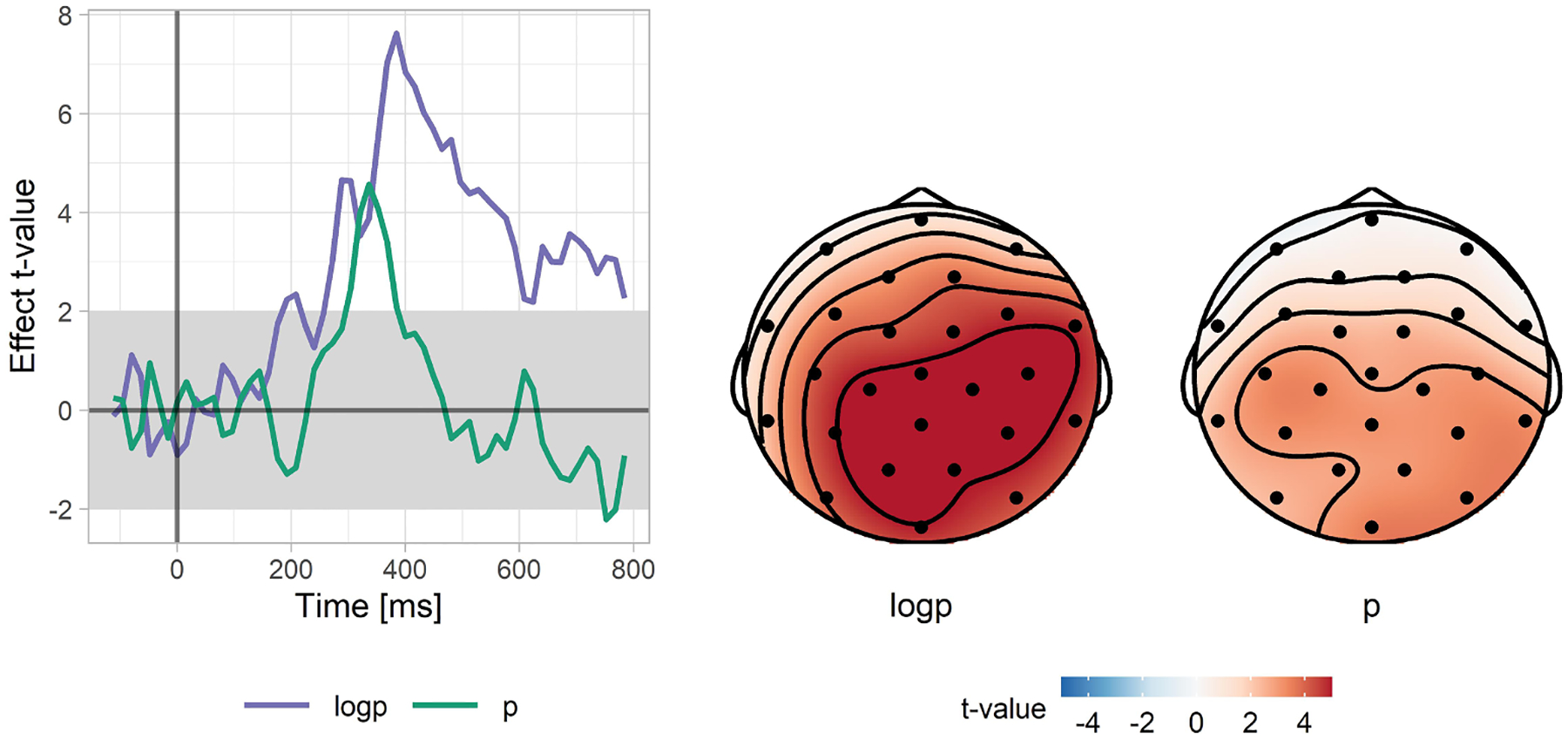
Unique contributions of the linear and logarithmic effects of word probability to ERPs. Note. The plots were generated via by-sample and by-electrode multiple mixed effects regressions testing unique contributions of the linear and logarithmic effects of word probability in models including both predictors. Left panel: t-values for the two predictors across time at a midline parietal electrode (MiPa); Right panel: scalp maps of averaged t-values for the linear and logarithmic effects in the 300–400 ms time-window.

In the previous analysis we focused on the whole N400 time-window. To further follow-up our finding of the statistically separable linear and logarithmic subcomponents of the N400 effect, we examined whether these effects have separable time-courses; in other words, we probed whether timing of these effects provides additional evidence for multiple influences of word probability on brain responses. For each electrode and for each time-point separately, we fitted a mixed effects regression model with the pre-word baseline, word probability, and log(word probability) as predictors. The model also included by-Subject and by-Item random intercepts and random slopes for the two probability-related effects, but no correlations between the random effects. This was an exploratory analysis in which we did not correct for multiple comparisons (however, we confirmed our findings in a subsequent replication analysis). The results are plotted in Fig. 6. As can be seen, the linear and logarithmic components have different time-courses. They both start at the about the same time (just before 300 ms), and both are present in the 300–400 ms time-window, but after that point the logarithmic contribution dominates. Inspection of the spatial distribution of both subcomponents in the 300–400 ms time-window, where both the linear and logarithmic components had a reliable influence, shows that the distribution of the two effects was very similar, with a medial, centro-parietal focus (typical of the N400; Van Petten & Luka, 2006). Thus, these analyses suggest that the two factors both capture variance in the N400 (and not some other ERP effect), but that the effect of log(p) is more sustained.

## Interim summary

The first part of our analysis brought a clear answer to the central question posed in the introduction. N400 amplitudes manifested a graded sensitivity to word probability across the full probability spectrum, extending into the range where human CPs are essentially at floor but where word probability differences can none-the-less be estimated from language models (here, GPT2). Interestingly, we found that the relationship between probability (whether estimated using human CPs or from the language model) and N400 amplitude was best fit using a linear function at the upper end of the probability spectrum, but was logarithmic in the range where human CPs are at or near 0. Therefore, in the second part of the analysis, we followed up on this observation by examining what kind of function best describes N400 amplitude patterns across the whole probability spectrum. Using a simple function in which the N400 was predicted as a weighted sum of word probability on the linear and on the logarithmic scale, we showed that each of the two functions explained unique variance that significantly improved the model’s fit. Looking timepoint by timepoint, we further showed that, although both functions explain variance beginning around 300 ms (i.e., around the time the N400 typically begins), the effect of linear word probability tapered off by about 400 ms whereas the effect of logarithmic word probability continued until at least 500 ms. The spatial distribution of the two effects, however, both resembled that typical of the N400. Thus, as we will detail in the General Discussion, although word probability on a linear and a logarithmic scale both affect the N400, these effects may derive from different sources.

First, however, we wanted to replicate this unexpected finding, especially since the differences between logarithmic and linear functions can be subtle and may be subject to overfitting (see also the discussion of this issue by Brothers & Kuperberg, 2021). This is particularly the case for the linear part, whose estimation relies on a fraction of the entire range of word probability on the logarithmic scale: The logarithmic effect was established in our study based on probabilities between log (–15) and log(0), whereas the linear effect was based on probabilities in the range log(−3) and log(0). Therefore, to test the robustness of the finding that both linear and log probability explain variance in the N400, we reanalyzed data from four other ERP datasets using similar materials and procedure. We checked if the logarithmic, linear, or linear + logarithmic model better fits the data. We focused on two time-windows that roughly correspond to the period in which both the linear and logarithmic effects are present (300–400 ms), and the period in which the logarithmic effect dominates (400–500 ms). In a final analysis, then, we combine the four new datasets and test the *predictive accuracy* of the linear, logarithmic and linear + logarithmic models—their ability to predict unseen observations. This is a far more stringent way of comparing models than testing the models’ fit to data because it circumvents the risk of overfitting to the current set of observations, resulting, for example, from a greater number of parameters used in the logp + p model.

## Replication analyses

### Methods

#### Materials

The dataset used for the replication analyses included data collected within four experiments (datasets 2–5 in Table 3).

**Fig. 7.**
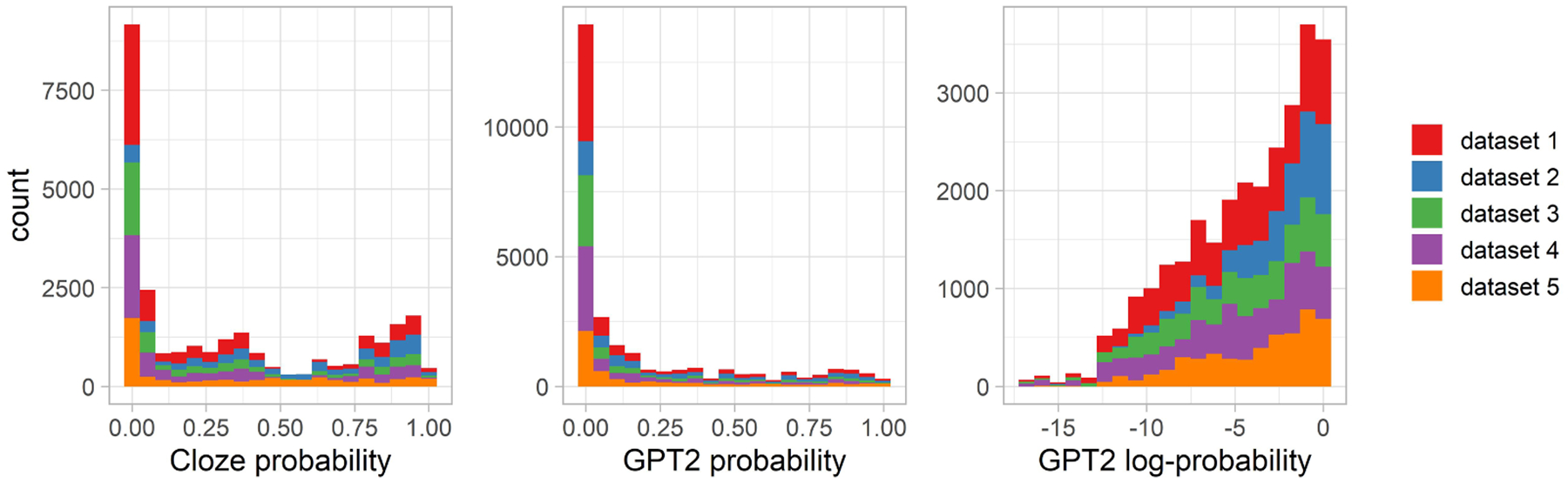
Histograms of the number of sentence endings with different word probabilities. Note. The three panels show different indices of word probability. Left panel: cloze probabilities; middle panel: GPT2-estimated probabilities; Right panel: GPT2-estimated log-probabilities. Note that each panel uses a different scale on the Y-axis.

Datasets 2–5 used very similar materials to those used in dataset 1. In fact, many of the sentences occurred in at least two datasets. Dataset 5 used sentences based on the items employed in the other datasets, but they were further modified by adding an adjective before the sentence ending. It used 168 unique sentence frames, each followed by either of 2 adjectives. Because the adjectives modified the CP of the following noun (sentence ending), each combination of a sentence frame and adjective is counted here as a separate item. Across all the five datasets, 347 unique sentence frames were used (but, as mentioned above, sometimes modified by adding an adjective). The left panel of Fig. 7 shows the CP distribution for sentence endings across the 5 datasets. As in dataset 1, unexpected endings (i.e., endings with zero or near-zero CP) were nevertheless plausible in their sentence contexts. The only exception is dataset 5, in which some combinations of nouns and adjectives could be considered implausible.

For each sentence frame and each sentence ending, we estimated the endings’ probability using the GPT2 language model, following the same procedure as described for Dataset 1. The middle and right panels of Fig. 7 show the distribution of GPT2-generated probability on the linear and the logarithmic scale. Overall, more probability mass (estimated using either human production norms or the language model) is focused on more expected words (with probability ≥ 0.05) than on less expected words (probability < 0.05; this equally applies to datasets 2–5 and to all datasets taken together).

#### Participants

Table 3 gives the numbers of participants entering into each dataset. Overall, participants were sampled from similar populations. The majority of them were university students (University of California, San Diego and University of Illinois at Urbana-Champaign), with fewer recruited from the general population. All participants were right-handed. Across the four datasets, 106 participants were analyzed (60 women, mean age 21 years, age range: 18–34 years).

#### Procedure

The experimental procedure was very similar across all five datasets (see above the Procedure section for the analysis of the first dataset). The only exception was dataset 4 in which the presentation of the sentences was divided into 8 blocks. Each block consisted of a study phase in which participants silently read the sentences for comprehension, a 30 s dis-tractor task (solving math problems), and a test phase, in which participants’ recognition memory for the sentence endings was tested. In the remaining experiments, a paper-and-pencil recognition test was conducted after the EEG session.

#### EEG recording

All experiments used 26 passive electrodes arranged in the same equidistant pattern (see Appendix A).

For uniformity, the data from each dataset were preprocessed in the same way (already described in the analysis of dataset 1), which may differ from the way in which these data had been analyzed in the original publications. The artifact correction procedure resulted in the removal of 7% of the data from Dataset 2, 8% of the data from Dataset 3, 7% of Dataset 4, and 6% of Dataset 5.

#### Statistical analyses

Our confirmatory analyses focused on pairwise comparisons between the logarithmic + linear model and two simpler models: linear-only and logarithmic-only. The comparisons were done in three time-windows: 300–500 ms (the full N400 time window), 300–400 ms (part of the N400 time-window in which linear word probability explained significant variance), and 400–500 ms (part of the N400 time-window in which logarithmic word probability was dominant). First, for each time-window we fitted three mixed-effects linear regression models. Next, we compared their log-likelihoods on the χ^2^ distribution. All comparisons were done while keeping the random effects structure equal (by-Subject and by-Item random intercepts and slopes for linear and logarithmic predictability). All models included the fixed effect for the pre-target-word baseline (Alday, 2019).

In addition to comparing the models’ fit to the data, we compared the models’ predictive accuracy. This metric addresses a potential problem associated with comparing models’ fit to the data. A model containing more parameters will always overfit more. Thus, finding a better fit of the more complex model combining linear and logarithmic effects does not necessarily indicate its superiority over simpler models containing either of the predictors. Predictive accuracy is immune to overfitting because overfitting to training samples will not increase fit to out-of-sample cases. However, this also comes at the cost of a much lower power to detect real differences between models of noisy data, such as psychophysiological recordings (see, e.g., Nicenboim et al., in preparation). The analysis of predictive accuracy mirrored the analysis of fit to the data, but, instead of LMER models, it compared a series of Bayesian models fitted using the brms package (Bürkner, 2017). For all predictors, we used regularizing Gaussian priors. The prior means for the GPT2.p, GPT2.logp, and baseline effects were set at their mean effect size in the analysis of dataset 1, with standard deviations equal to half the effect size. In this way, the priors are in the general ballpark of realistic effect sizes for each predictor, but they allow for a large variation in effect sizes, including cases in which there is no effect. In this way, we restrict the prior to have a good sign (word probability has a positive correlation with the N400 amplitude) and to permit a broadly plausible range of values, without imposing any extra knowledge, as would be the case if we narrowly centered our priors on specific effect sizes obtained from dataset 1. A wide prior is also important because the posterior distribution of effect sizes will shift toward larger values for models including either GPT2.p or GPT.logp (compared to the model including both), due to the removal of the correlated predictor. The covariates were standardized and for them we used regularizing priors (we did not know what values the coefficients would take in a multiple regression including all the other predictors). The priors were centered at zero with standard deviation set such that a change of predictor value by 1SD should maximally lead to a change in N400 amplitude by +/− 2 μV. All prior distributions are presented in Table 4.

Models were fitted using 63,000 sampling iterations (collected across 14 chains). Predictive accuracy was tested by measuring differences in expected log-pointwise predictive density (elpd) between the models, using the Pareto-smoother leave-one-out cross-validation (PSIS-LOO; Vehtari et al., 2021). Predictive density for problematic observations (Pareto *k* > 0.7) was obtained by refitting the models.

Finally, to get an estimate of differences in the size of the logarithmic and linear effects in the 300–500 ms time-window across the five datasets, we fitted a Bayesian model predicting the N400 amplitude analogous to the model described above, but also including a simple effect of dataset, as well as interactions of dataset with GPT2.p and GPT2.logp. The priors for the simple effects of dataset were set at N(0, 1.5), for the interactions of dataset and GPT2.p at N(0, 1.3), and for interactions of dataset and GPT2.logp at N(0, 0.15). In other words, priors for the interactions, in conjunction with the priors for simple effects of GPT2.p and GPT2.logp, were consistent with datasets getting a broad range of positive predictability effects, also including no effect of predictability.

## Results

### Comparisons of models’ fit to data

**Fig. 8.**
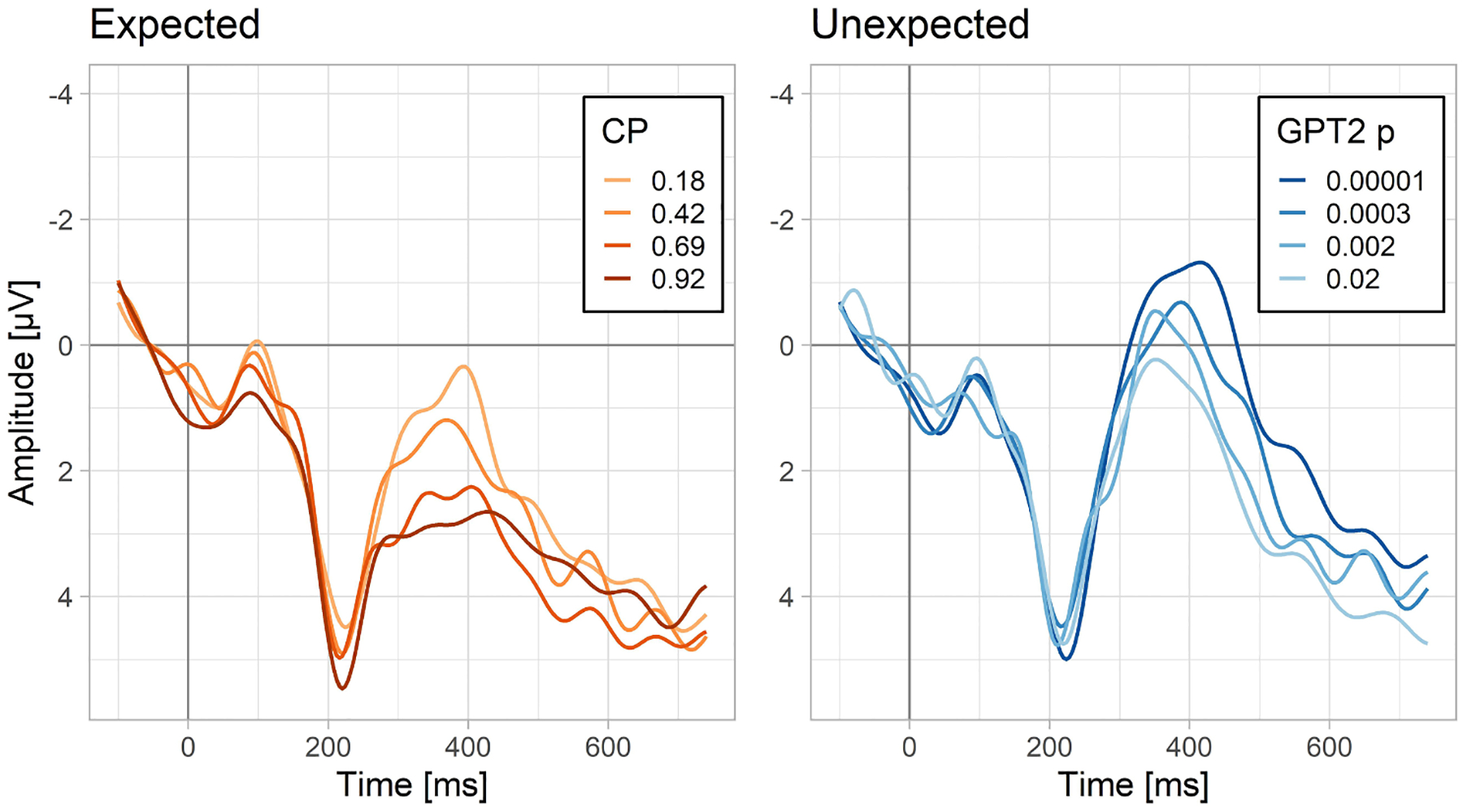
ERPs to Expected and Unexpected Sentence Endings in Datasets 2–5. Note. ERPs were recorded at a midline parietal electrode (MiPa). Left panel: expected endings; right panel: unexpected endings. The ERPs are broken down by target word probability grouped into four bins with an equal number of elements, estimated using CP tests (left panel) and GPT2 log-probability (right panel; values in the legend were exponentiated to linear probability). The Y-scale is the same as in Fig. 4. However, the bins are different due to difference in item composition between the Datasets 1 and 2–5. For display purposes, the ERPs were low-pass filtered at 12 Hz.

Just as in Dataset 1, word predictability led to a robust modulation of the N400 both in expected and unexpected words (Fig. 8) in the analysis of the new datasets.

**Fig. 9.**
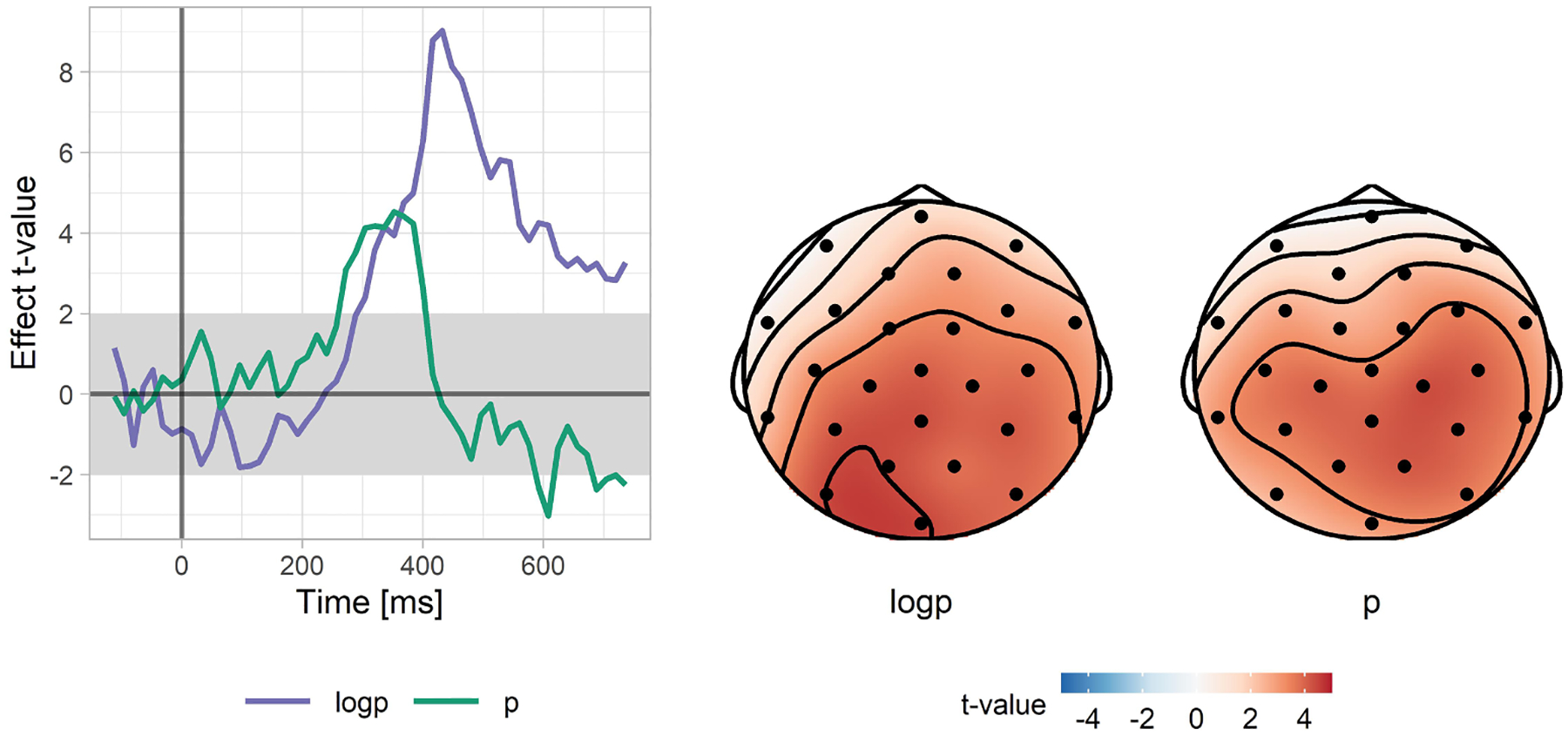
Unique contributions of the linear and logarithmic effects of word probability to ERPs in Datasets 2–5. Note. The plots were generated via by-sample and by-electrode multiple mixed effects regressions testing unique contributions of the linear and logarithmic effects of word probability in models including both predictors. Left panel: t-values for the two predictors across time at a midline parietal electrode (MiPa); Right panel: scalp maps of averaged t-values for the linear and logarithmic effects in the 300–400 ms time-window.

In the replication analysis we were particularly interested in whether these additional datasets would also provide evidence that the linear and logarithmic effects each explain variance on top of the other word-probability-related predictor. Model comparisons robustly replicated the first analysis, with both the linear and logarithmic parts being present in the 300–400 ms time-window, but with only the logarithmic part present in the 400–500 ms time-window (see Table 5). In the analysis of the entire N400 time-window (300–500 ms), both effects are significant, although the linear effect contributes less to explaining N400 amplitudes (see also Fig. 9, which presents the outcome of a point-by-point exploratory analysis analogous to the analysis presented in Fig. 6).

Note that in all the analyses conducted here, we focused on the impact of predictability on the N400 effect. Predictability also affected scalp locations and time-windows associated with the P600 and Anterior Positivity effects, both of which are beyond the scope of this work. For curious readers, in Appendix F we visualize the time-course of the effect of predictability across all electrodes.

### Test of the models’ predictive accuracy

We compared the differences in predictive accuracy between the three models of N400 amplitude: linear predictability only, logarithmic predictability only, and logarithmic and linear predictability combined. Table 6 shows the results of the comparison. In the 300–400 ms time-window, the combined model shows a modest advantage in predictive accuracy over the p-only model, and a more substantial advantage over the logp-only model. In both the 400–500 ms and the 300–500 ms time-windows, models including logarithmic predictability are more predictive than the model including only linear predictability. In summary, the analysis of leave-one-out predictive accuracy shows that in the 300–500 ms and 400–500 ms adding the linear component does not improve the models’ ability to predict unseen cases. In contrast, the linear component improves the out-of-sample predictions in the 300–400 ms time-window.

### Comparison of the effect sizes across the datasets

**Fig. 10.**
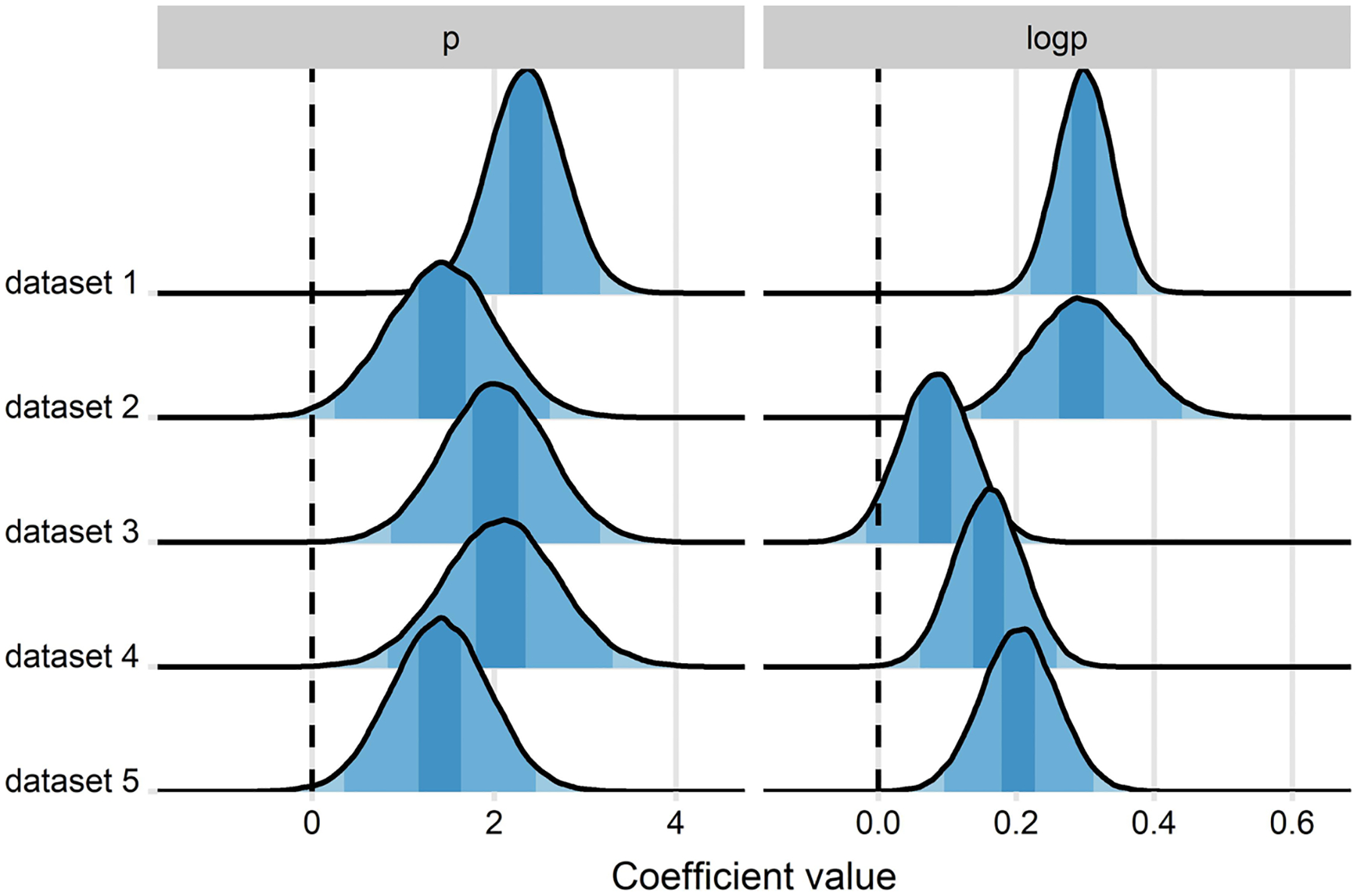
Posterior distribution of the linear and the logarithmic effects of word probability fitted in a Bayesian mixed effects model of the amplitude in the 300–400 ms time-window.

Fig. 10 shows a comparison between the sizes of the linear and the logarithmic effects across the datasets from a Bayesian mixed-effects model of amplitudes in the 300–400 ms time-window (where both effects were prominent). The magnitude of the linear effect was relatively constant, whereas the magnitude of the logarithmic effect varied more across datasets. This suggests that the magnitude of the logarithmic effect may be dependent on participants’ processing strategy, the composition of items, or other factors related to task length and task demands (e.g., passive reading vs. reading to succeed on periodic memory tests). See Appendix D for a full model summary.

## General discussion

In this study, we asked about the scope of context-based facilitation in language comprehension. The various variants of the surprisal account (Aurnhammer & Frank, 2019; Kuperberg & Jaeger, 2016; Levy, 2008; Smith & Levy, 2013) posit that context effects extend to all words in the lexicon and bear a logarithmic-shaped relationship to word probability. Alternative accounts (see discussion in Brothers & Kuperberg, 2021) instead hypothesize that the comprehension system allocates activation to likely next words in proportion to their probability, creating a linear relationship between word probability and contextual facilitation. On this account, then, contextual facilitation is effectively limited in scope to words that are relatively probable in the input. In this study, we set out to test these views by examining whether context effects follow a linear or logarithmic pattern.

We reanalyzed a series of experiments in which participants read for comprehension short sentences whose endings could be either expected or unexpected but plausible. We measured mean amplitudes of the N400 component evoked by the sentence endings. The N400 arises at a critical period in word processing when the perceptual form of the input makes contact with distributed knowledge in semantic memory—i.e., when that input is undergoing “semantic access”. Critically, this process is exquisitely sensitive to the state of the semantic system, as shaped by prior processing of the context. In particular, N400 amplitudes are reduced to the extent that some of the information that would normally be evoked by an input has already been activated in the course of processing the prior context (Kutas & Federmeier, 2011). The N400 thus can serve as a highly sensitive metric of the extent to which semantic processing of an incoming word is facilitated in a given context. We measured the predictability of sentence endings in two ways. First, we used human-generated CP norming, which is a source of the best-known estimates of expected words’ predictability. We also used a state-of-the-art neural language model (GPT-2) trained at the next-word prediction task, which, according to recent evidence, is very successful in predicting brain activity and reading behavior, although its outputs have never been directly compared against human production norms. In addition to providing comparative estimates of the predictability of expected words in the sentences, the model also provided us with fine-grained indices of differences in the predictability of unexpected words (for most of which CP tests indicated zero predictability). We were interested if these fine-grained differences in predictability could explain variance in N400 amplitude to the unexpected endings. We were also interested in the shape of the relationship (linear vs. logarithmic) between the offline indices of predictability and the N400.

We found that for expected sentence endings, although not as good a predictor as CP, the language model was successful at explaining N400 amplitude patterns to the expected sentence endings. Critically, we found that for unexpected words, where CPs are unable to account for any variation in the N400, the language model explained an even greater range of N400 amplitudes than it did in the case of expected words. Moreover, we showed that, whether assessed based on CPs or on model-generated probabilities, the relationship between word predictability and the N400 for expected words is linear. In contrast, for the unexpected words, the relationship is logarithmic. Finally, we showed that a function including both the linear and the logarithmic components is able to describe the N400 values across the whole range of probabilities. An analysis testing the contribution of both components at each time-point showed that they are both prominent in the 300–400 ms time-window, but, in a later time-window, the linear component tapers off and only the logarithmic component remains. In subsequent analyses we confirmed that in the 300–400 ms time-window, the model consisting of both subcomponents offers a superior fit as well as superior predictive accuracy, compared to models consisting of only the linear or the logarithmic subcomponents. We discuss each of these findings in detail below, followed by a consideration of the implications of these findings for our understanding of the mechanisms involved in comprehension.

### GPT-2 as a substitute for cloze probability

CP tests have been the most pervasive and well-established ways of measuring word predictability. They are also one of the strongest known predictors of reading behavior and indices of brain activity occurring during language comprehension. To validate the GPT-2 language model as a tool for estimating word predictability, we focused on expected items (i.e., items with CP not at or near zero) and compared GPT-2′s estimates of word probability with those obtained via the cloze procedure. We found that both tools seemed to capture the same phenomenon, as evidenced by the strong correlation between them and the fact that they explained the same variance in the N400 brain response to words. Human-derived estimates provided a better fit to N400 amplitudes than the GPT2 model. The highest discrepancies between the two estimates of word predictability occurred for sentence endings with the highest CP, which were often estimated as less predictable by the model. Nevertheless, the fact that GPT2 predicts variations of N400 amplitude in congruent words is impressive given that our sentences were constructed to prohibit simple predictions about the sentence endings based on word-to-word associations: Prediction of the sentence endings was possible only via the extraction of higher-order information encoded in the context. The advantage of CPs may stem from information inaccessible to language models (which are trained on purely linguistic material), such as the grounding of language in other modalities, conceptual understanding of the world, and experience with the events described by the sentences. Nevertheless, GPT2-like language models appear to be a good alternative to cloze tests in estimating the predictability of words in coherent sentences, especially when probabilities need to be estimated at scale—for example, for all words in presented sentences instead of just selected target words. They are also superior for estimation of predictability of non-obligatory parts of the sentence, such as modifiers (see, e.g., Szewczyk et al., 2021), which generally tend to have low CP (and it is even possible that language models will eventually provide better predictors of the N400 than the cloze procedure, c.f. Michaelov et al., 2021). Finally, as we will discuss next, they permit estimates of the probabilities of words that might never or only rarely be produced in CP norms.

### Contextual facilitation is graded and extends to very unpredictable words

A core aim of the study was to test if N400 amplitudes to unexpected words – i.e., words that, although plausible, are very unlikely to be produced in a given context – are nevertheless sensitive to the degree of their (un)predictability. This is the area in which CP tests offer no useful predictions due to lack of variance in their estimates. We found that, indeed, the N400 to unexpected words is acutely sensitive to their (log) probability. In fact, across the 5 datasets, the range of N400 amplitudes explainable by words’ probability in the unexpected part of the scale was 1.5 times larger than the case of expected words (see also Fig. 5 for an estimate based on dataset 1). In principle, this range could be even wider because the unexpected words in the current study were selected to be always at least possible to use in their sentence contexts, so we did not test truly anomalous words, which would probably have even lower log-probability and lead to even greater N400 effects. Past studies employing the traditional CP index were able to measure only the tip of the iceberg of contextual facilitation on the N400.

Our findings also highlight that for the brain, predictability is a continuum. Even though prior work has divided experimental items into “congruent” and “incongruent”, it appears that overall (with the exception of linear effects that show up for the most predictable words; see below), there are no qualitative differences between N400s to congruent and incongruent words on top of the graded effect of predictability.

In the past, there were a few attempts to manipulate the degree of unpredictability/implausibility of words presented in sentence contexts (approximated by controlling plausibility, violating animacy in addition to incongruity, etc.; see Introduction for a review). These studies yielded mixed results; although some studies showed that the degree of unpredictability affects the N400, several other showed that it does not. One possible explanation of this inconsistency is related to the fact that sometimes the degree of unpredictability was based on a subjective estimation of plausibility by a normative group of participants, which may be a poor approximation of the fit of the word in a given context. When we used plausibility estimates to explain the N400 to unexpected words across 4 datasets^4^, this predictor did not improve the model’s fit over and above a model containing GPT2 log-probabilities. Human decision processes may simply have relatively little access to the activation states of semantic representations, rendering such explicit judgments inaccurate.

Overall, these findings suggest that context-based facilitation is graded and not restricted to linearly predictable words. The amount of facilitation increases with every new bit of information about the upcoming word contained in the representation of context. For the estimation of the amount of context-based facilitation, language models, such as GPT2, may be an indispensable tool.

### The effect of contextual facilitation is both linear and logarithmic

In the models fitted separately to expected and unexpected words we found that expected words were better described using a linear function of probability, whereas unexpected items are better described using a logarithmic function. Critically, this outcome was maintained even when both the linear and logarithmic functions were based on probabilities coming from the same source – the GPT2 language model. This removes potential criticism whereby human-based CP tests measure probability on a different scale than computer-based language models (see Introduction).

When we tried to model both expected and unexpected words using the same function, we found that the logarithm of word probability roughly predicts the N400 to a dataset consisting of both expected and unexpected words. However, when the model was enriched with a predictor corresponding to the word’s predictability on the linear scale, the model’s fit to the data improved. This initial finding on dataset 1 was replicated on four other datasets. In addition to looking at the models’ fit to the data, we tested the models’ predictive accuracy across the four replication datasets using leave-one-out cross-validation. Despite being a much more conservative test, this analysis showed that the more complex model makes better predictions about novel datapoints, addressing a potential concern that the better fit of the composite function resulted from overfitting. Finally, the different time-courses of both factors (linear occurring only in the 300–400 ms time-window, logarithmic effect occurring in the entire N400 time-window) further attest to their separable influence. Therefore, in the following discussion, we build on the hypothesis that the improved predictability of the model consisting of both the linear and logarithmic effects is not just an advantage arising from using an increased number of predictors, which inadvertently affords more flexibility on modeling a function’s shape. Instead, we will posit that these effects arise from two dissociable (but temporally overlapping) influences on language comprehension.

Our finding that both logarithmic and linear functions of probability predict N400 amplitudes speaks to a recent discussion in the literature about the relationship between predictability and indices of context-based facilitation (Brothers & Kuperberg, 2021; Smith & Levy, 2013; see also Yan et al., 2017). According to the proportional pre-activation account, lexical features of upcoming words get hierarchically (pre) activated from higher cortical representations, in line with a hierarchical generative framework described by Kuperberg and colleagues (2020). The account assumes that passing activations from higher to lower cortical levels is costly and bounded by a limited pool of metabolic resources. The account proposes that a rational way of allocating these resources is to activate the words’ features in linear proportion to the probability of encountering these words in the upcoming input. Consistent with this view and with some prior studies examining cloze probability effects on the N400 (DeLong et al., 2005; Kutas & Hillyard, 1984; Nieuwland et al., 2018), we found that N400 amplitudes to expected words *did* in fact track linear word probability. This effect had its greatest force within the first part of the N400 time window. However, contrary to this view, we also found that there was substantial N400 amplitude variance that could be explained by probability within the unexpected items. These items had CPs around 0 and the variability in N400 evoked by these words tracked probability on the log scale.

The logarithmic relationship is better accounted for by the surprisal account. Levy (2008; see also Kuperberg & Jaeger, 2016) proposes that the language comprehension system maintains and, with each word, updates a probability distribution over different parses/message-level representations of the current sentence. These representations are to a varying degree consistent with different upcoming words and the amount of processing necessary when an upcoming word is presented is proportional to the word’s surprisal. Alternatively, Aurnhammer and Frank (2019) proposed that the logarithmic relationship results from the distance (i.e., Kullback-Leibler divergence) between the distribution of probability over all words before the target word is apprehended and the distribution after the word has been processed, in which all probability mass is focused on the target word only. Finally, Smith and Levy (2013) explained the logarithmic relationship by showing that if words are analyzed incrementally, in fragments, and the overall facilitation for a word consists of the sum of predictability effects for these fragments, then the net effect of word predictability will be logarithmic, regardless of the function linking the probability of the individual fragments and the resulting facilitation.

Consistent with these accounts, we found substantial variance in N400 amplitudes in the range where CP is at or near 0, predicted by word probability estimates from GPT2 and following a log scale. These effects were present in the entire N400 time-window but dominated in its later part and overall, they are responsible for the bulk of the N400 effect. This finding is incompatible with theories/models assuming a linear relationship between word probability and indices of contextual facilitation (Brothers & Kuperberg, 2021; Fitz & Chang, 2019). However, the additional linear effect that we observed is inconsistent with the claims of surprisal theories, especially with its strongest variant (Levy, 2008), which posits that surprisal acts as a causal bottleneck—that is, that the entire effort related to apprehension of a word manifests only through log-shaped effects.

In sum, although some aspects of each view were supported by the data, overall the data pattern is inconsistent with either view. What does this more complex pattern suggest about the mechanisms underlying context effects? We argue that consideration of the functional properties of the N400 in combination with the observed data pattern offers some ideas about the processing that takes place during word apprehension.

### The N400 reflects both semantic and “lexical” context-based effects

Decades of research on the N400 strongly suggest that the effect relies on conceptual representations that go beyond word stimuli. The N400 is elicited by any potentially meaningful stimulus: words in all modalities as well as “word-like” stimuli (pseudowords and letter strings) with no learned meaning (e.g., Holcomb et al., 2002; Kutas & Hillyard, 1980; Laszlo & Federmeier, 2007), as well as non-linguistic stimuli, including pictures (e.g., Ganis et al., 1996; Proverbio & Riva, 2009), gestures (e.g., Özyürek et al., 2007), and environmental sounds (e.g., Orgs et al., 2007), in verbal and non-verbal contexts alike. In all these situations, the N400 reflects the amount of new semantic information becoming active in response to current input (Federmeier, 2022; Kutas & Federmeier, 2000, 2011; Federmeier & Laszlo, 2009). The conceptual (as opposed to lexical) character of the N400 is further supported by studies manipulating the degree of stimulus overlap with the prior context on the featural level. For example, in the related-anomaly paradigm, implausible and lexically unexpected words elicit a reduced N400 effect, as long as they share semantic features with the strongly expected word (Federmeier & Kutas, 1999; see Rommers et al., 2013 for a related finding)^5^.

In the context of our findings, we propose that the degree of featural overlap on the conceptual level covaries with the logarithmic part of the word probability effect. The logarithmic relationship with word probability results from the properties of the mapping between probabilities of words and the level of contextual support for semantic features corresponding to these words. More specifically, each concept denoted by a word can be decomposed into many semantic features, some of which may be activated in advance over the course of processing a sentence (for example, the higher-level representation of the sentence can include features of likely upcoming concepts). However, it will usually be the case that only some of these features are required to uniquely identify a word (e.g., if we know that an upcoming concept refers to a pet that barks, we don’t need the additional information that it has a tail and fur to be highly certain that it will be denoted by the word “dog”). According to our account, the N400 is exquisitely sensitive to semantic feature activation levels, such that, based on differences in the extent of feature overlap, contextually unexpected words will vary in the N400 amplitude they elicit in a manner that can be captured by small differences in word probability estimated by GPT-2. However, although each additional overlapping feature between the context and an incoming word will cause a linear decrease in N400 amplitude, the pool of compatible words will decrease exponentially, leading to an exponential increase in word probability and, in consequence, the logarithmic relationship between probability and N400 (*log*(*p*) *N*400 and *p exp*(*N*400) are equivalent)^6^. Formally, our account is equivalent to the explanation proposed by Smith and Levy (2013), if one substitutes word fragments with semantic features. In the modified version, conditional word probability depends on the conditional probabilities of associated semantic features:

$$p\left(\text{word}|\text{context}\right)=p_{1}\times \cdots \times p_{k}$$

(where p_1_ … p_k_ correspond to probabilities of k semantic features given the context), and the processing cost (e.g., N400 amplitude) depends on the sum of a function of feature probability:

$$\text{processing cost}=f\left(p_{1}\right)+\cdots +f\left(p_{k}\right)$$

Smith and Levy (2013) showed that for a very high *k*, the processing cost becomes proportional to −*log*(*p*_*word*_). For some possible functions *f*, the relationship between word probability and processing cost becomes logarithmic only when *k* ≫ 10, so within the original proposal, the word would have to be incrementally analyzed in many (tens, possibly hundreds) fragments. This would be more fragments than there are typically phonemes in any word, which raises the question of the identity of these fragments, and, overall, makes the original proposal implausible. However, if we assume that instead of word fragments word processing costs result from activation of *k* semantic features (and all features always have some residual activation; i.e., their activation is never zero), then a very high *k* value becomes natural.

What, then, is the source of the linear part of the relationship between N400 and word probability? We posit that the linear part of the N400 effect is linked to semantic representations of the *specific* word or word sense that has been made highly likely by the context. The lexical character of these representations is suggested by the fact that the magnitude of the effect directly corresponds to the probability of specific words, while their semantic (as opposed to, for example, orthographic or phonological) character is implied by the timing of the effect—i.e. occurring after 200–300 ms, when word-form-related effects affect the ERPs (e.g. Carreiras et al., 2009; Chauncey et al., 2008; Hauk et al., 2009; Holcomb & Grainger, 2006; Laszlo & Federmeier, 2014). We propose that word forms are associated with a set of semantic features. These features get coactivated whenever the word is encountered and soon become interconnected, forming bundles reflecting the meaning of the word^7^. When the representation of the context is strong and structured enough, it coalesces into these lexical-semantic representations which then become point attractors in a multidimensional semantic space. When the context activates some features related to the word, the bundling between the features leads to the recovery of the complete pattern of all features that are associated with the word. Not all of these features are necessarily associated with the context but all of them get activated when the word is encountered. This differentiates the linear effect from the logarithmic effect. Conceptual representation of the context consists of features that are temporarily bound, for example, as a result of reading a sentence. The logarithmic effect arises when some of these features happen to overlap with the features of an upcoming word. In contrast, the linear effect emerges when the contextual activation is specific enough that it triggers the activation of the structured semantic representation of the word, which may include many word features that are irrelevant in the current context. These context-irrelevant features would become activated anyway upon encountering the word. However, when not only contextually-relevant, but also contextually-irrelevant features become activated prior to the word’s occurrence, an even greater reduction of the N400 amplitude takes place once the word eventually appears, which is why linear predictability affects the N400 amplitude beyond the effect of logarithmic predictability.

Nieuwland et al. (2020) also observed that amplitude variance in the N400 time window was explained by multiple factors, with different time courses. In particular, they found that word predictability, as assessed by CP, explained variance between approximately 250–450 ms whereas plausibility ratings explained variance beginning around 350 ms and continuing beyond the N400 time window until the end of the recorded epoch (1000 ms). Based on differences in the types of processes presumed to be linked to word predictability versus plausibility ratings, Nieuwland et al. suggested that there are multiple distinct types of mechanisms at work in the N400 time window. In our analyses, we found that linear word predictability, as measured by CP, affected N400 amplitudes beginning around 250 ms. In our analysis of incongruent items, however, we found that plausibility did not significantly explain variance over and above log probability estimated from GPT-2. Thus, we find that word predictability remains a driving factor throughout the N400 time window, albeit changing in scale from linear to log based on what we theorize to be differences in which (sets of) semantic features are active (see also Footnote 6). Other influences could indeed affect later parts of the waveform, but that is outside the scope of the present investigation.

We would like to stress that even though it is possible to dissociate factors that influence amplitude variation in the N400 time-window into linear and logarithmic aspects of predictability (in our dataset) or into CP and plausibility ratings (in Nieuwland et al., 2020), we view these as all comprising parts of the same N400 response, corresponding to the same cognitive process: perception-driven first-pass access to the meaning of a stimulus in its context. The N400 effect subsumes synchronized activity of a distributed brain network, whose specific configuration depends on the semantics of any particular stimulus. For example, according to the hub-and-spoke theory (Lambon Ralph et al., 2017; Patterson et al., 2007), the activated network includes both common areas (i.e., the ATL) and meaning-specific areas that are different for each word (see, e.g., Deniz et al., 2019; Huth et al., 2016). But even within the semantic hub, the specific region that is activated may be different depending on the semantics of the stimulus (Bonner & Price, 2013; Kochari et al., 2021; Lau, Weber, et al., 2016; X. Wang et al., 2019). Moreover, different brain regions may be recruited in a temporally cascaded way within the broad N400 time-window (see, e.g., Amsel et al., 2013; Toneva et al., 2020). As we have shown here, parts of this activity may have a more linear or more logarithmic relationship to word predictability. Yet this entire cascaded process that recruits and synchronizes different brain areas is *invariably* fired whenever our senses apprehend a meaningful stimulus. That is, the function of interest, semantic access, arises through time and via the interaction of distributed brain areas, which is why it makes sense to treat the N400 – and the impact on it of word probability, with both its linear and logarithmic components – as a functional whole.

A final question concerns the source of the differences in the time-courses between the linear and logarithmic effects. To address this question, we will assume that during semantic access word representations are not merely looked up in semantic memory, but rather constructed and aligned with the context (Elman, 2009; Federmeier & Laszlo, 2009). Under this assumption, the shorter time-course of the linear effect can be explained simply by the fact that the effect is based on the activation of contextually irrelevant features. Such features do not contribute to building a higher-level semantic representation that is aligned with the context, so that only the contextually-relevant features continue to play a part in further shaping the context-adjusted representation of the word meaning.

## Summary

In this paper we have shown that contextual support is not limited to linearly predictable words: Every bit of semantic feature overlap between the context and an incoming word makes N400 amplitude to that word more positive. This happens even in the case of words that are highly unpredictable and thus unlikely to ever be used in a given context. Furthermore, we have shown that, overall, the relationship between word probability and the N400 is logarithmic, but that in the first half of the N400 time window (~300–400 ms), there is also a linear impact of word probability. We proposed that both the linear and the logarithmic components correspond to the amount of new semantic information becoming active. The logarithmic component reflects the reduction of N400 amplitude due to overlap with the semantic features that have been activated as part of the representation of the context. The linear component reflects additional reduction in N400 amplitude due to overlap with bundled semantic features that are associated with (part of the conceptual representation of) a given word (but not with the context) and that have been jointly activated due to high contextual support for that given word. Although here we have focused on internal models built from sentences and their impact on word processing, words are, of course, just one of many routes to the representations of meaning. Given the domain generality of the N400, it is likely that these findings are broadly applicable and inform how internal models facilitate perception in other domains. Humans sample reality by looking for meaningful patterns in information catered by their senses, creating representations of words, visual objects, scenes, faces, and so on. Semantic information associated with these familiar patterns thus comes to be bundled, and thus they all, in their own way, structure semantic memory. This structuring, in turn, becomes part of the mind’s model of the world, which guides the course by which further stimuli are apprehended.

## Supplementary Material

Apeendices

## Figures and Tables

**Table 1:** Likelihood Comparison Between Models of the N400 to Expected Sentence Endings.

Comparison	Δ logLik	χ^2^	p
CP: lin vs. log			
p vs p + logp	0.5	0.96	.33
logp vs p + logp	1.5	3.08	.08
GPT2: lin vs. log			
p vs p + logp	0.0	0.02	.89
logp vs p + logp	7.1	14.22	.001
linear: CP vs. GPT2			
GPT2 vs GPT2 + CP	13.8	27.67	.000001
CP vs GPT2 + CP	0.9	0.07	.79

*Note:* logLik is an index of each model’s fit to the data and is used to obtain the χ^2^ value. χ^2^ and p are used to obtain the results of the likelihood ratio test between the compared models. For example, the values in the first row give the result of the likelihood ratio test comparing the p-only model with the p + logp model (testing if adding logp predictor significantly improves the fit of the model that already contains p predictor).

**Table 2:** Linear mixed effects model of N400 amplitudes to expected and unexpected words, including both the linear and logarithmic predictors of word predictability.

Effect	Estimate	SE	t	by-Item SD	by-Subject SD
Intercept	−4.33	0.48	−9	0.73	2.66
Baseline	0.48	0.02	21.8	0.05	0.10
Log-frequency (std)	0.31	0.14	2.2	0	0.42
Concreteness (std)	0.36	0.11	−3.2	0.39	0.22
OLD20 (std)	0.2	0.13	1.6	0.47	0.31
Word position (std)	0.45	0.18	2.6	–	0.81
GPT2 p	1.43	0.5	2.9	1.28	0.75
GPT2 log(p)	0.33	0.04	8.3	0	0.09

*Note*: Abbreviations: std – standardized; residual SD = 7.34.

**Table 3:** Overview of sample sizes of all studies reanalyzed in this study.

Dataset No	Reference	N subjects	N items	N datapoints
1	Federmeier et al. (2007)	32	282	7856
2	Wlotko & Federmeier (2012)	16	300	4440
3	Unpublished dataset	26	300	4855
4	Hubbard et al. (2019)	32	192	5705
5	Szewczyk et al. (2021)	32	336*	4939

*Note:* Dataset 1 was already analyzed in the previous section. For an explanation of how the items were counted in dataset 5, see below in text.

* Dataset 3 also included filler sentences and sentences ending with synonyms of the most expected word, and these were not used in the reanalysis reported below.

**Table 4:** Priors used in the baseline Bayesian mixed effects models.

Prior name	Prior
Intercept	N(0,3)
Baseline	N(0.5,0.25)
OLD20 (std)	N(0,2)
Log-frequency (std)	N(0,2)
Word position (std)	N(0,2)
Concreteness (std)	N(0,2)
GPT2 probability	N(2.6,1.3)
GPT2 log-probabilty	N(0.3,0.15)
Residual SD	N(10,3)
SD in Random effects	N(0,2)

**Table 5:** Results of Model Comparisons Between Linear, Logarithmic, and Linear + Logarithmic Linear Mixed Effects Models for Datasets 2–5.

comparison	Δ logLik	χ^2^	P
300–400 ms			
p vs p + logp	11.2	22.4	< .000001
logp vs p + logp	15	30.1	< .000001
400–500 ms			
p vs p + logp	33.8	67.6	< .000001
logp vs p + logp	0	0	.85
300–500 ms			
p vs p + logp	25.9	51.9	< .000001
logp vs p + logp	5.4	10.8	< .01

*Note*. All predictabilities were estimated using the GPT-2 model.

**Table 6:** Comparison of Leave-One-Out Predictive Accuracy Between Models Including Linear, Logarithmic and Linear + Logarithmic Predictability.

Model	Difference in elpd	Difference SE
300–400 ms		
p + logp		
p	−7	3.6
logp	−9.2	4.6
400–500 ms		
logp		
p + logp	−0.8	0.6
p	−19.8	6.2
300–500 ms		
p + logp		
Logp	−2.5	3.0
P	−15.0	5.1

*Note:* In each time-window, models are ordered from the model with the highest expected log-predictive density (elpd). The highest-scoring model is used as a baseline for the comparison of elpd with the other two models.

## Footnotes

1 We use the term “facilitation” broadly to mean not only faster behavioral responses (in manual RT or eyetracking paradigms) but also reduced activation in brain measures (as is common in neuroimaging); in the latter case, facilitation (e.g., in an ERP measure) may, but does not have to, be directly reflected in any specific behavioral measure that might be collected during comprehension.

2 However, the addition of a quadratic term *did* improve fit between CPs and N400s measured to words using visual half-field presentation to bias processing to the contralateral hemisphere. Wlotko and Federmeier (2013) thus concluded that the linear relationship between CP and N400 amplitude in central vision is an emergent property of the combination of multiple processing mechanisms.

3 We omit here studies using the related-anomaly paradigm (e.g., Federmeier & Kutas, 1999) or studies showing various sorts of “semantic illusions” (see Bornkessel-Schlesewsky & Schlesewsky, 2008; Kuperberg, 2007 for reviews), wherein patterns of facilitation are known to be dissociated from offline measures of word predictability or plausibility.

4 We did not collect plausibility data for Dataset 5.

5 However, we also note that there is modeling work assuming that the N400 is directly linked to lexical instead of semantic representations (Fitz & Chang, 2019).

6 This account is similar to the explanation of logarithmic effects proposed by Kuperberg and Jaeger (2015; which extends to conceptual processing the proposal by Levy, 2008) in that both accounts assume that the effects are generated at the conceptual level. However, in contrast to the other account, we do not posit that the N400 necessarily reflects updating of higher-level semantic representations (e.g., updating of the situation model; see also Nieuwland et al., 2020; Rabovsky et al., 2018). Instead, we claim that evidence so far can be parsimoniously explained by the costs of activating semantic features. For example, reductions of N400 amplitude can result from activations that are unrelated to the situation model (see, e.g., Rommers & Federmeier, 2018; Szewczyk & Schriefers, 2018).

7 For simplicity, we assume here that each word is associated with only one meaning. However, our explanation naturally extends to situations in which a word form is associated with many senses, under the assumption that semantic access depends on both the word form and the representation of the context, as is typically assumed by recurrent models of language comprehension, such as the Retrieval-Integration model (Brouwer et al., 2017). In such a scenario, only some word senses compatible with the word form *and* the context may attract activation.
