# Supplementary material for: Context-based facilitation of semantic access follows both logarithmic and linear functions of stimulus probability
Source: J Mem Lang. Author manuscript; Available in PMC 2022 Nov 3. (PMC9631957; doi:10.1016/j.jml.2021.104311)
Supplement: Apeendices [file NIHMS1812024-supplement-Apeendices.docx]

# Appendix A. Approximate locations and the label of each electrode channel.


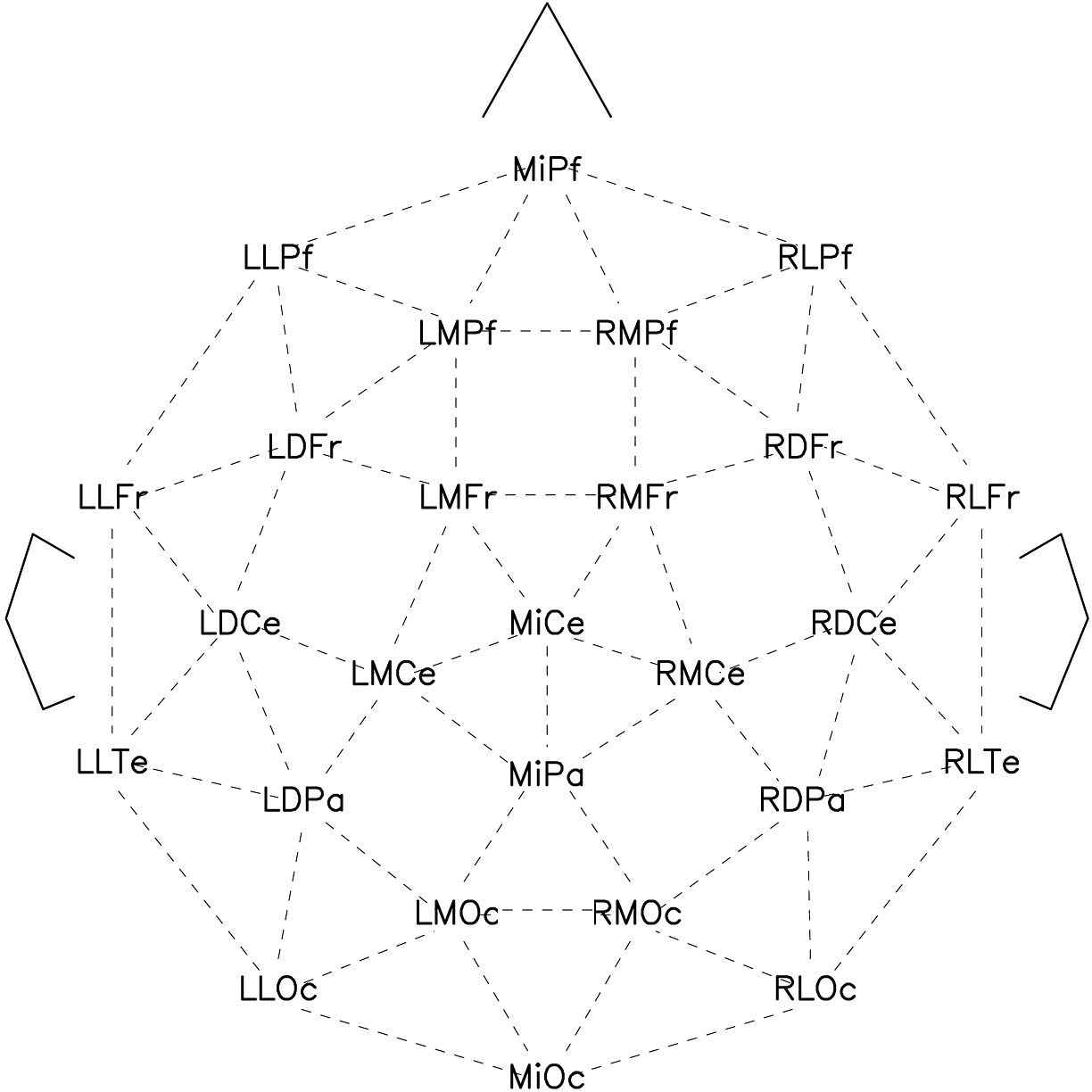


# Appendix B. Model for incongruent words, dataset 1.

**Table B.**

*Linear mixed-effects model of N400 amplitude in unexpected endings in Dataset 1, using pre-word baseline and GPT2 log-probability as predictors, as well as four covariates.*

| Effect | Estimate | SE | t | by-Item | by-Subject |
| --- | --- | --- | --- | --- | --- |
|  |  |  |  | SD | SD |
| Intercept | -5.66 | 0.5 | -11.4 | 0.77 | 2.72 |
| Baseline | 0.49 | 0.02 | 20.1 | 0.02 | 0.12 |
| GPT2 log(p) | 0.25 | 0.05 | 5.1 | - | 0 |
| Log-frequency (std) | 0.35 | 0.18 | 1.89 | - | 0.54 |
| OLD20 (std) | 0.19 | 0.15 | 1.25 | - | 0 |
| Concreteness (std) | -0.73 | 0.13 | -5.45 | - | 0 |
| Position (std) | 0.20 | 0.18 | 1.15 | - | 0.73 |

# Appendix C. Models of the N400 amplitude in incongruent words with plausibility as a predictor (dataset 1).

Below is a summary of a GAM model predicting the N400 amplitude to unexpected items. The model contains plausibility both as a parametric effect and within a smooth term, to separate its linear and non-linear parts. The model is fit using the following formula:

n400 ~ s(Subject, bs = "re") + s(Item, bs = "re") + c_bline + s(c_bline, bs = "cr") + s(Subject, c_bline, bs = "re") + s(Item, c_bline, bs = "re") + plausibility + s(plausibility, bs = "cr") + s(Subject, plausibility, bs = "re") + s_logfreq + s(s_logfreq, bs = "cr") + s(Subject, s_logfreq, bs = "re") + s_concr + s(s_concr, bs = "cr") + s(Subject, s_concr, bs = "re") + s_old20 + s(s_old20, bs = "cr") + s(Subject, s_old20, bs = "re") + s_pos_start + s(s_pos_start, bs = "cr") + s(Subject, s_pos_start, bs = "re")

Parametric coefficients:

Estimate Std. Error t value Pr(>|t|)

(Intercept) -4.71123 0.59990 -7.853 5.36e-15 ***

c_bline 6.47563 0.74110 8.738 < 2e-16 ***

plausibility -0.39118 0.12813 -3.053 0.00228 **

s_logfreq 0.50247 0.18933 2.654 0.00799 **

s_concr -0.85212 0.16985 -5.017 5.52e-07 ***

s_old20 0.09652 0.15661 0.616 0.53771

s_pos_start 0.36543 0.24005 1.522 0.12802

---

Signif. codes: 0 ‘***’ 0.001 ‘**’ 0.01 ‘*’ 0.05 ‘.’ 0.1 ‘ ’ 1

Approximate significance of smooth terms:

edf Ref.df F p-value

s(Subject) 28.3298820 31.0000000 28.071 < 2e-16 ***

s(Item) 37.0339384 252.0000000 0.174 0.031852 *

s(c_bline) 3.3208436 4.2323201 4.598 0.000771 ***

s(Subject,c_bline) 20.8017521 31.0000000 5.817 0.00000666 ***

s(Item,c_bline) 0.0005474 257.0000000 0.000 0.624945

s(plausibility) 0.0003500 0.0006440 0.383 0.987479

s(Subject,plausibility) 3.2052563 31.0000000 0.944 0.429365

s(s_logfreq) 0.2528216 0.4329054 0.561 0.622305

s(Subject,s_logfreq) 12.1069209 31.0000000 0.651 0.015989 *

s(s_concr) 0.3178009 0.5301232 0.647 0.558141

s(Subject,s_concr) 0.0004353 31.0000000 0.000 0.535310

s(s_old20) 0.0001100 0.0002021 0.245 0.994387

s(Subject,s_old20) 0.1350832 31.0000000 0.004 0.459279

s(s_pos_start) 0.8778244 1.2927179 1.404 0.370669

s(Subject,s_pos_start) 16.3648991 31.0000000 1.192 0.000289 ***

---

Signif. codes: 0 ‘***’ 0.001 ‘**’ 0.01 ‘*’ 0.05 ‘.’ 0.1 ‘ ’ 1

In the above model, the plausibility smooth term does not shows up as significant, but the parametric (linear) effect of plausibility does. As noted in the main text, even though the parametric effect of plausibility is significant, it is a worse predictor of the N400 amplitude in unexpected items than GPT2 log-probability.

# Appendix D. Summary of the Bayesian mixed-effects model comparing the magnitude of the linear and logarithmic effects of word probability in a model of amplitude in the 300-400ms time-window across the 5 datasets.

Family: gaussian

Links: mu = identity; sigma = identity

Formula: n400 ~ c_bline + c_gpt2_p + c_gpt2_logp + dset_12 + dset_13 + dset_14 + dset_15 + c_gpt2_p:(dset_12 + dset_13 + dset_14 + dset_15) + c_gpt2_logp:(dset_12 + dset_13 + dset_14 + dset_15) + s_logfreq + s_concr + s_pos_start + s_old20 + (1 + c_gpt2_p + c_gpt2_logp + c_bline + s_logfreq + s_concr + s_pos_start + s_old20 || Subject) + (1 + c_gpt2_p + c_gpt2_logp + c_bline + c_gpt2_p:(dset_12 + dset_13 + dset_14 + dset_15) + c_gpt2_logp:(dset_12 + dset_13 + dset_14 + dset_15) || Item)

Data: d5 (Number of observations: 27762)

Samples: 14 chains, each with iter = 6000; warmup = 1500; thin = 1;

total post-warmup samples = 63000

Group-Level Effects:

~Item (Number of levels: 663)

Estimate Est.Error l-95% CI u-95% CI Rhat Bulk_ESS Tail_ESS

sd(Intercept) 0.78 0.08 0.62 0.93 1.00 25537 36454

sd(c_gpt2_p) 0.46 0.34 0.02 1.25 1.00 12290 19336

sd(c_gpt2_logp) 0.18 0.03 0.12 0.23 1.00 15781 22279

sd(c_bline) 0.04 0.01 0.01 0.06 1.00 10124 8838

sd(c_gpt2_p:dset_12) 0.73 0.53 0.03 1.97 1.00 20940 32300

sd(c_gpt2_p:dset_13) 1.07 0.71 0.05 2.60 1.00 17223 27079

sd(c_gpt2_p:dset_14) 0.86 0.61 0.04 2.26 1.00 22123 30051

sd(c_gpt2_p:dset_15) 0.58 0.43 0.02 1.61 1.00 28801 31084

sd(c_gpt2_logp:dset_12) 0.21 0.10 0.02 0.39 1.00 8044 12644

sd(c_gpt2_logp:dset_13) 0.06 0.05 0.00 0.17 1.00 21251 30111

sd(c_gpt2_logp:dset_14) 0.05 0.04 0.00 0.13 1.00 26906 32131

sd(c_gpt2_logp:dset_15) 0.06 0.04 0.00 0.16 1.00 26666 32480

~Subject (Number of levels: 138)

Estimate Est.Error l-95% CI u-95% CI Rhat Bulk_ESS Tail_ESS

sd(Intercept) 2.27 0.15 2.00 2.59 1.00 14568 26094

sd(c_gpt2_p) 0.65 0.39 0.04 1.42 1.00 10651 23555

sd(c_gpt2_logp) 0.15 0.02 0.10 0.19 1.00 21689 30579

sd(c_bline) 0.13 0.01 0.11 0.15 1.00 22056 37118

sd(s_logfreq) 0.27 0.10 0.04 0.45 1.00 12075 13596

sd(s_concr) 0.12 0.08 0.01 0.30 1.00 18329 28985

sd(s_pos_start) 0.68 0.07 0.54 0.83 1.00 30566 46087

sd(s_old20) 0.11 0.08 0.00 0.28 1.00 20435 28060

Population-Level Effects:

Estimate Est.Error l-95% CI u-95% CI Rhat Bulk_ESS Tail_ESS

Intercept -3.16 0.37 -3.88 -2.43 1.00 9357 18464

c_bline 0.52 0.01 0.49 0.54 1.00 20902 34308

c_gpt2_p 2.35 0.41 1.55 3.16 1.00 47201 47758

c_gpt2_logp 0.30 0.04 0.22 0.38 1.00 37508 45067

dset_12 0.99 0.63 -0.28 2.22 1.00 11914 21122

dset_13 0.45 0.56 -0.64 1.53 1.00 10529 19771

dset_14 0.97 0.53 -0.09 2.00 1.00 9879 19287

dset_15 2.60 0.53 1.54 3.63 1.00 11107 21969

s_logfreq 0.33 0.08 0.18 0.48 1.00 65200 53696

s_concr -0.32 0.07 -0.46 -0.19 1.00 64596 53448

s_pos_start 0.47 0.09 0.30 0.64 1.00 45015 48416

s_old20 0.20 0.07 0.06 0.33 1.00 70065 53192

c_gpt2_p:dset_12 -0.92 0.63 -2.16 0.31 1.00 65658 50277

c_gpt2_p:dset_13 -0.33 0.62 -1.55 0.88 1.00 73815 52493

c_gpt2_p:dset_14 -0.28 0.66 -1.57 1.01 1.00 72824 53356

c_gpt2_p:dset_15 -0.95 0.62 -2.17 0.28 1.00 60592 50753

c_gpt2_logp:dset_12 -0.00 0.08 -0.15 0.15 1.00 61565 51410

c_gpt2_logp:dset_13 -0.21 0.06 -0.33 -0.10 1.00 49331 49799

c_gpt2_logp:dset_14 -0.14 0.06 -0.25 -0.03 1.00 48488 48609

c_gpt2_logp:dset_15 -0.09 0.06 -0.22 0.03 1.00 51090 51750

Family Specific Parameters:

Estimate Est.Error l-95% CI u-95% CI Rhat Bulk_ESS Tail_ESS

sigma 7.99 0.04 7.92 8.06 1.00 100649 44976

# Appendix E. Package versions used in the analyses and data processing

> sessionInfo()

R version 4.0.3 (2020-10-10)

Platform: x86_64-w64-mingw32/x64 (64-bit)

Running under: Windows 10 x64 (build 19043)

Matrix products: default

locale:

[1] LC_COLLATE=English_United States.1252 LC_CTYPE=English_United States.1252

[3] LC_MONETARY=English_United States.1252 LC_NUMERIC=C

[5] LC_TIME=English_United States.1252

system code page: 1250

attached base packages:

[1] parallel stats graphics grDevices utils datasets methods base

other attached packages:

[1] car_3.0-10 carData_3.0-4 loo_2.4.1 scales_1.1.1

[5] piecewiseSEM_2.1.2 ggridges_0.5.3 brms_2.15.0 Rcpp_1.0.6

[9] doParallel_1.0.16 iterators_1.0.13 foreach_1.5.1 itsadug_2.4

[13] plotfunctions_1.4 mgcv_1.8-33 nlme_3.1-149 lme4_1.1-26

[17] Matrix_1.2-18 eegUtils_0.5.0 RColorBrewer_1.1-2 patchwork_1.1.1

[21] ggplot2_3.3.3 stringr_1.4.0 dplyr_1.0.5 data.table_1.14.0

[25] eeguana_0.1.5.9000

loaded via a namespace (and not attached):

[1] readxl_1.3.1 backports_1.2.1 plyr_1.8.6 igraph_1.2.6

[5] lazyeval_0.2.2 shinydashboard_0.7.1 splines_4.0.3 listenv_0.8.0

[9] crosstalk_1.1.1 TH.data_1.0-10 rstantools_2.1.1 inline_0.3.17

[13] digest_0.6.27 htmltools_0.5.1.1 viridis_0.5.1 rsconnect_0.8.16

[17] fansi_0.4.2 checkmate_2.0.0 magrittr_2.0.1 openxlsx_4.2.3

[21] globals_0.14.0 RcppParallel_5.0.3 matrixStats_0.58.0 R.utils_2.10.1

[25] xts_0.12.1 sandwich_3.0-0 beepr_1.3 prettyunits_1.1.1

[29] colorspace_2.0-0 signal_0.7-6 haven_2.3.1 xfun_0.22

[33] callr_3.5.1 crayon_1.4.1 jsonlite_1.7.2 R.matlab_3.6.2

[37] survival_3.2-7 zoo_1.8-9 glue_1.4.2 gtable_0.3.0

[41] emmeans_1.5.5-1 V8_3.4.0 pkgbuild_1.2.0 rstan_2.21.2

[45] future.apply_1.7.0 abind_1.4-5 mvtnorm_1.1-1 DBI_1.1.1

[49] edfReader_1.2.1 miniUI_0.1.1.1 viridisLite_0.3.0 xtable_1.8-4

[53] foreign_0.8-80 stats4_4.0.3 StanHeaders_2.21.0-7 DT_0.17

[57] httr_1.4.2 htmlwidgets_1.5.3 threejs_0.3.3 DiagrammeR_1.0.6.1

[61] ellipsis_0.3.1 pkgconfig_2.0.3 R.methodsS3_1.8.1 utf8_1.2.1

[65] tidyselect_1.1.0 rlang_0.4.10 reshape2_1.4.4 later_1.1.0.1

[69] munsell_0.5.0 cellranger_1.1.0 tools_4.0.3 visNetwork_2.0.9

[73] cli_2.3.1 generics_0.1.0 audio_0.1-7 fastmap_1.1.0

[77] yaml_2.2.1 processx_3.5.0 zip_2.1.1 purrr_0.3.4

[81] future_1.21.0 mime_0.10 projpred_2.0.2 R.oo_1.24.0

[85] pracma_2.3.3 compiler_4.0.3 bayesplot_1.8.0 shinythemes_1.2.0

[89] plotly_4.9.3 curl_4.3 gamm4_0.2-6 tibble_3.1.0

[93] statmod_1.4.35 stringi_1.5.3 ps_1.6.0 Brobdingnag_1.2-6

[97] forcats_0.5.1 lattice_0.20-41 nloptr_1.2.2.2 markdown_1.1

[101] shinyjs_2.0.0 vctrs_0.3.6 pillar_1.5.1 lifecycle_1.0.0

[105] bridgesampling_1.0-0 ini_0.3.1 estimability_1.3 httpuv_1.5.5

[109] R6_2.5.0 promises_1.2.0.1 gridExtra_2.3 rio_0.5.26

[113] parallelly_1.24.0 codetools_0.2-16 boot_1.3-25 colourpicker_1.1.0

[117] MASS_7.3-53 gtools_3.8.2 assertthat_0.2.1 withr_2.4.1

[121] shinystan_2.5.0 multcomp_1.4-16 hms_1.0.0 grid_4.0.3

[125] tidyr_1.1.3 coda_0.19-4 minqa_1.2.4 shiny_1.6.0

[129] base64enc_0.1-3 dygraphs_1.1.1.6 tinytex_0.30

# Appendix F. The time-course of the effect of predictability across all electrodes

**Figure F.**

*The ERPs and massive tests of the effect of word log-predictability, based on data from all datasets.*


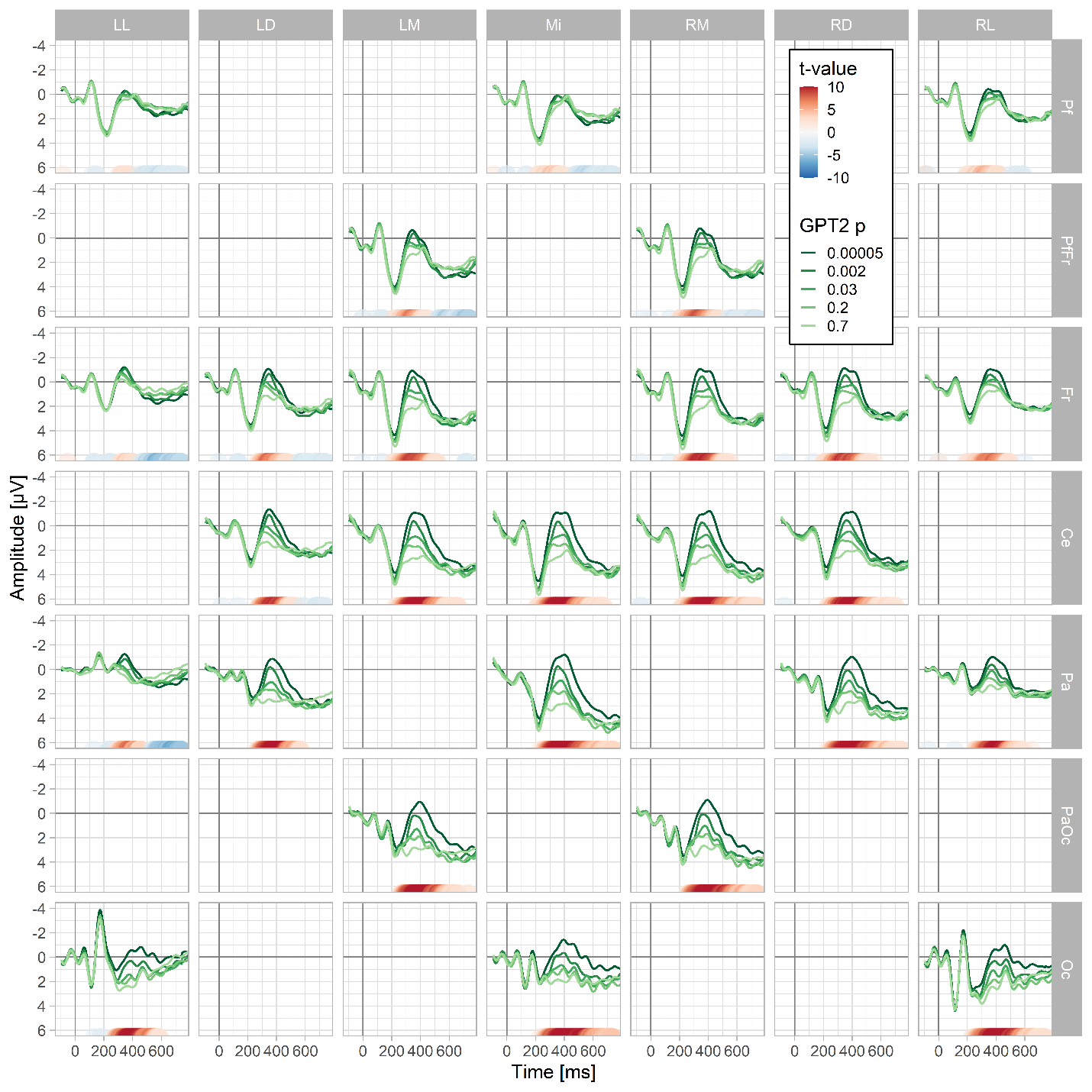


*Note.* The ERPs are broken down by target word probability estimated using GPT2 log-probability (across expected and unexpected words) grouped into five bins with an equal number of elements. Probability values in the legend were exponentiated to linear probability. The blue-red color scale at the bottom of each panel shows the t-value of GPT2 log-probability effect coming from a mixed model conducted at each electrode and time-point. t-values with absolute size smaller than 2 are not plotted. For display purposes, the ERPs were low-pass filtered at 12Hz.

**Appendix G. Additional exploratory tests related to plausibility**

**Figure G1.**

*Scatterplots of the relationship between plausibility and GPT2 log-probability.*


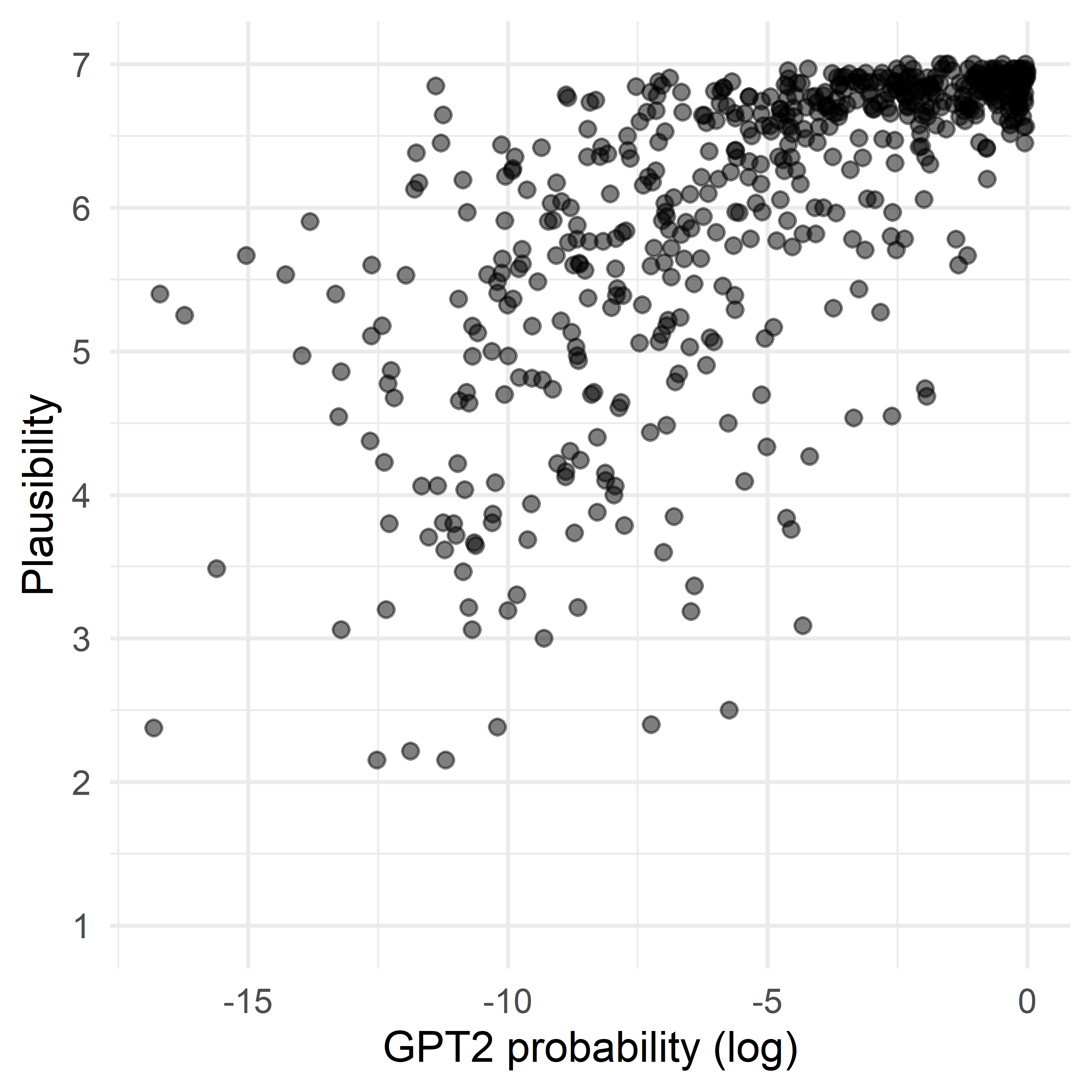


The overall correlation between log-probability and plausibility was r=.67 (when tested separately for expected and unexpected items, the correlation coefficients were r=.32 and r=.39, respectively).

**Figure G2.**

*ERPs to Expected and Unexpected Sentence Endings split into plausibility bins.*


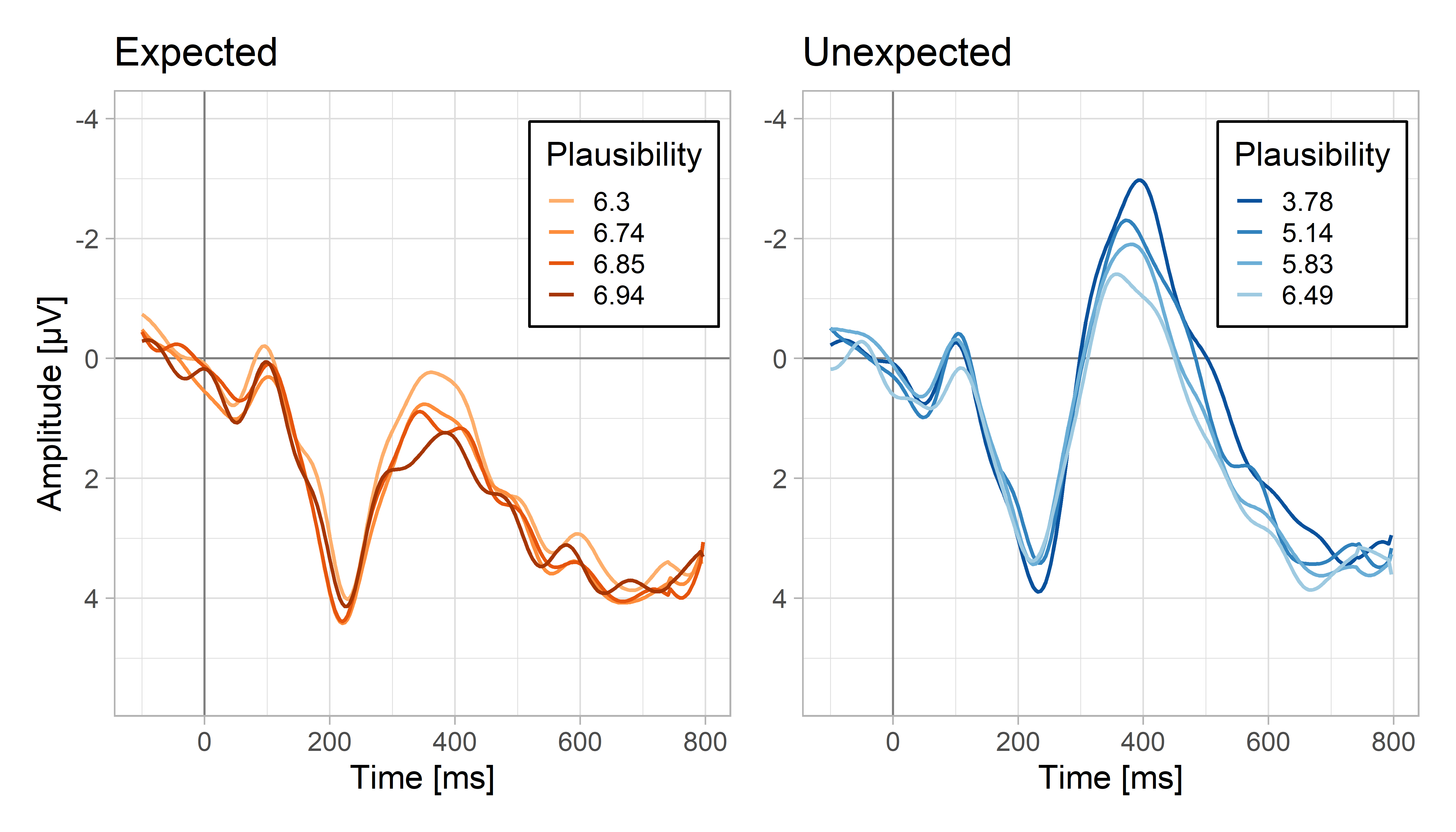


*Note.* ERPs were recorded at a midline parietal electrode (MiPa). Left panel: expected endings; right panel: unexpected endings. The ERPs are broken down by target word plausibility grouped into four bins with an equal number of elements. The ERPs were low-pass filtered at 12Hz. ERPs are based on Datasets 1-4.
